# Association Between Albuminuria and Glomerular Filtration Rate With Incident Frailty

**DOI:** 10.1016/j.ekir.2024.11.017

**Published:** 2024-11-18

**Authors:** Elisa K. Bongetti, Anna L. Wilkinson, James B. Wetmore, Anne M. Murray, Robyn L. Woods, Sara Espinoza, Michael E. Ernst, Michelle A. Fravel, Suzanne G. Orchard, Le Thi Phuong Thao, Joanne Ryan, Rory Wolfe, Kevan R. Polkinghorne

**Affiliations:** 1 Department of Nephrology, Monash Medical Centre, Monash Health, Melbourne, Victoria, Australia; 2 Department of Medicine, Monash University, Melbourne, Victoria, Australia; 3 School of Public Health and Preventive Medicine, Monash University, Melbourne, Victoria, Australia; 4 Disease Elimination, Burnet Institute, Melbourne, Victoria, Australia; 5 Melbourne School of Population and Global Health, University of Melbourne, Victoria, Australia; 6 Division of Nephrology, Hennepin Healthcare, Minneapolis, Minnesota, USA; 7 Berman Center for Outcomes and Clinical Research and Department of Medicine, Hennepin Healthcare Research Institute, and Department of Medicine, Geriatrics Division, Hennepin Healthcare Minneapolis, Minnesota, USA; 8 Center for Translational Geroscience, Department of Medicine, Cedars-Sinai Medical Center, Los Angeles, California, USA; 9 Department of Pharmacy Practice and Science, College of Pharmacy; and, Department of Family Medicine, Carver College of Medicine. The University of Iowa, Iowa City, Iowa, USA

**Keywords:** albuminuria, chronic kidney disease, elderly, frailty, kidney function tests, older adult

## Abstract

**Introduction:**

The association between estimated glomerular filtration rate (eGFR) and albuminuria with incident frailty in generally healthy older individuals is unclear. We investigated whether baseline eGFR or urine albumin-to-creatinine ratio (UACR) are associated with incident frailty and assessed them using 2 separate methods: a modified Fried frailty phenotype (FP), and a deficit accumulation frailty index (FI).

**Methods:**

This was a secondary analysis of the ASPirin in Reducing Events in the Elderly (ASPREE) randomized trial cohort, which enrolled independent, healthy, older adults aged ≥65 years in Australia and the USA. Time to incident frailty was analyzed using discrete time survival analysis.

**Results:**

In total, 16,965 participants were included using the FP, mean age was 75.0 ± 4.5 years, median baseline eGFR was 78.6 ml/min per 1.73 m^2^ (interquartile range [IQR]: 67.6–89.5), and the median UACR was 0.80 mg/mmol (0.50–1.50). Data to generate the FI outcomes were available for 12,272 participants. The relationships between eGFR and both incident FP and FI were nonlinear, such that an eGFR < 30 or ≥ 95 ml/min per 1.73 m^2^ was significantly associated with an increased risk of incident frailty. For every doubling of baseline UACR, risk of incident frailty increased by 4% using the FP (hazard ratio [HR]: 1.04, 95% CI: 1.02, 1.07) and the FI (HR: 1.04, 95% confidence interval [CI]: 1.01–1.07).

**Conclusion:**

In older, generally healthy adults, both low and high eGFRs were associated with increased risk of incident frailty. Doubling of UACR, even at low levels, was independently associated with incident frailty.

## Graphical abstract

**Figure:**
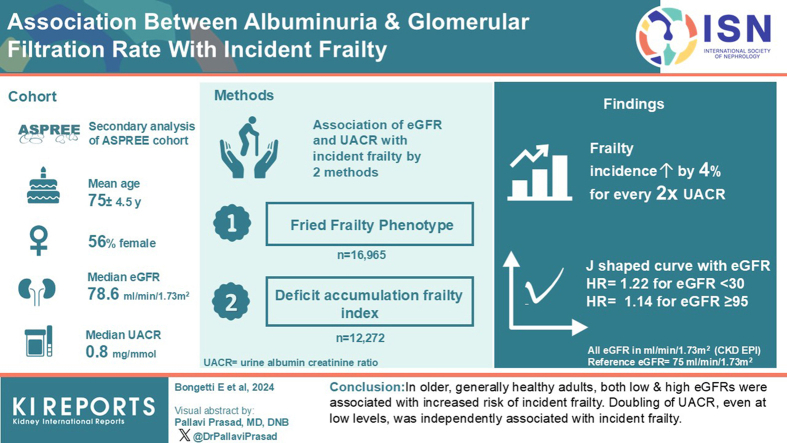

Frailty is an age-related state of reduced physiological reserve characterized by increased vulnerability to stressors, hospitalizations, and increased risk of death.^1^, ^2^, ^3^ Identifying risk factors for frailty, particularly biomarkers collected during routine clinical practice, may facilitate early diagnosis, intervention, and conceivably, preservation of functional status.^4^^,^^5^ Chronic kidney disease (CKD) and frailty are frequently comorbid and independently associated with decreased quality of life and increased mortality.^6^^,^^7^ Frailty is a recognized state of accelerated aging typified by declining health across multiple domains, including decreased physical and cognitive performance.^8^, ^9^, ^10^ Accordingly, frailty is highly common in patients with advanced CKD and end-stage kidney disease on dialysis.^11^ However, the longitudinal risk of developing frailty in otherwise healthy, older individuals with mild abnormalities in markers of kidney function is unclear.

The two markers of kidney function frequently obtained in routine clinical practice are serum creatinine (used to calculate eGFR) and UACR. There is an extensive body of literature on the association of eGFR and health outcomes, much of which has focused on individuals with moderate to severe CKD.^12^, ^13^, ^14^ However, perhaps paradoxically, in adults aged >60 years, preserved creatinine-based eGFR has been associated with negative health outcomes, including increased mortality.^15^ In this instance, elevated eGFR may not reflect true preservation in GFR, particularly as most eGFR equations are validated in younger populations and can overestimate kidney function in older adults.^16^^,^^17^ In addition, reduced skeletal muscle mass is an important non-GFR determinant of reduced serum creatinine, which is frequently observed in older adults with sarcopenia and frailty.^18^, ^19^, ^20^, ^21^, ^22^ Although elevated creatinine-based eGFRs are known to cooccur with preexisting frailty,^22^ there is limited evidence on whether elevations in creatinine-based eGFR in nonfrail, independently-living, older adults is a risk factor for incident frailty.

Few studies have been conducted on the association of albuminuria and incident frailty, particularly on relatively low levels of albuminuria in generally healthy older individuals. Just as serum creatinine, and therefore eGFR, may be reflective of factors other than kidney function, albuminuria is a marker of impaired endothelial function and inflammation, and low level albuminuria is predictive of cardiovascular disease, dementia, and mortality.^23^^,^^24^ Data from cross-sectional and small-scale longitudinal studies have demonstrated that albuminuria, even at very low levels, is associated with frailty.^3^^,^^25^ However, the risk of incident frailty in independent, older individuals with low-level albuminuria, based on data from large, longitudinal studies has not been determined.

The aim of this study was to investigate whether creatinine-based eGFR and/or albuminuria are associated with incident frailty in initially healthy older adults. Previous evidence suggests that low eGFR (i.e., CKD) is associated with frailty, and that high creatinine-based eGFR may occur in the setting of muscle loss in older adults, and therefore might be a risk for frailty.^16^^,^^17^^,^^26^ Therefore, we hypothesized that there would be a nonlinear J-shaped association between baseline eGFR and longitudinal incident frailty, such that both high (≥ 90 ml/min per 1.73 m^2^) and low (< 45 ml/min per 1.73 m^2^) eGFR would be associated with an increased risk of incident frailty. We also hypothesized that increased baseline albuminuria, independent of eGFR, would be associated with increased risk of incident frailty.

## Methods

### Study Design and Population

This was a secondary analysis of the ASPREE study cohort, whose methodology has been extensively described and reported previously.^27^ In short, ASPREE was a double-blind randomized, placebo-controlled primary prevention trial designed to determine whether daily 100 mg enteric-coated aspirin extends physical disability-free and dementia-free life (so-called disability-free survival), in a healthy older population. The trial enrolled 19,114 community dwelling (87% in Australia and 13% in the USA) adults who were not on aspirin for secondary prevention and were free of many major health problems, such as cardiovascular disease, dementia, independence-limiting physical disability, or any illness expected to limit their life expectancy to less than 5 years. Eligible participants were aged ≥70 years, except for African American or Hispanic participants who were eligible at age ≥65 years. For this analysis, participants who were frail at baseline, or were missing baseline frailty or kidney function data (i.e., absence of either eGFR or UACR) were excluded. ASPREE was approved by multiple institutional review boards in the USA and Australia, registered with International Standard Randomized Controlled Trial Number Register (ISRCTN83772183) and clinicaltrials.gov (NCT01038583), and undertaken in accordance with the Declaration of Helsinki. All participants provided written informed consent.

### Exposure and Study Measures

The exposures were baseline levels of 2 measures of kidney function: albuminuria, measured as a spot UACR in mg/mmol; and creatinine-based eGFR, estimated with the CKD Epidemiology Collaboration 2021 equation (CKD-EPI_2021_).^28^ Further details are provided in the Supplementary Methods.

Study measures included participant demographic variables and health measures, assessed at trial enrolment. These included age (years), sex at birth (male, female), body mass index (in kg/m^2^), ASPREE study drug treatment allocation (aspirin or placebo), smoking history (current, former, never), alcohol consumption history (current, former, never), years of education (< 12 or ≥ 12 years), diabetes history (defined as a self-reported, fasting blood glucose level > 126 mg/dl or treatment for diabetes), mean systolic and diastolic blood pressure (mm Hg), polypharmacy (defined as 5 or more prescription medications), country of residence (Australia or USA), and dyslipidemia (defined as those taking cholesterol-lowering medications or serum cholesterol ≥ 5.5 mmol/l in Australia or ≥ 6.2 mmol/l in the USA).

### Frailty Outcome

Time to incident frailty was defined as the annual visit at which the participant first met the criteria for frailty, or in the absence of frailty, was censored at their last annual visit. We considered frailty as defined by 2 frailty scales: a modified version of the Fried FP,^29^ and the deficit accumulation FI, which have previously been used with the ASPREE cohort.^30^^,^^31^ Use of the FP and the FI in separate, parallel analyses was undertaken with the intention of providing a complementary and robust assessment of frailty. Although the FP and the FI both characterize frailty, each scale has different criteria and limitations.^7^ The FP defines 5 physical components: unintentional weight loss, slowness, exhaustion, weakness, and low activity.^29^ The FI is a composite score, including up to 67 terms across physical, cognitive, and medical domains.^31^ Details on how the scores were derived from the ASPREE data, and the definitions of frailty and prefrailty according to each criterion, are included in the Supplementary Methods.

In separate models for FP and the FI, frailty was dichotomized as ”frail” or “not frail.” Given that frailty is a dynamic state, only the time from baseline to an individual’s first occurrence of frailty was considered in this analysis. Prefrailty, which describes an intermediate state between nonfrail and frailty, significantly increases risk of developing future frailty.^32^ To control for the effect of baseline prefrailty on longitudinal frailty risk, baseline prefrailty was included as a covariate in the analyses.

### Statistical Analysis

All statistical analyses were undertaken in R version 4 (R Core Team, 2022) and Stata MP 17 (Stat Corp, College Station, TX). UACR was log_2_-transformed for the analysis to manage the positive skew of the variable and enhance interpretability of regression coefficients for this variable. eGFR was analyzed as a continuous and categorical variable (eGFR <45, 45–59, 60–89, ≥90 ml/min per 1.73 m^2^) in separate models. Descriptive statistics were presented as means and SDs for symmetrically distributed continuous variables, median and IQR for skewed continuous variables, and absolute numbers and percentages for categorical variables.

### Missing Data

#### Fried FP

As per the ASPREE protocol, the collection of all relevant FP data items was not conducted at every year of follow-up; therefore, it was anticipated there would be missing longitudinal data for this variable (Supplementary Table S1). All FP data items were collected at participants’ milestone visit in the final year of the study, which could have been conducted as the annual visit at any year from year 3 onward, depending on when an individual was recruited to the study. To manage this structural missing data, longitudinal missing frailty data was imputed using multivariate imputation by chained equations with the *mice* package in R and under missing at random assumptions. Imputation of missing values minimizes the risk of introducing selection bias, which can occur when participants with missing values are excluded.^33^ A description of the imputation methodology is presented in Supplementary Table S2. All variables to be included in the final analysis models were used in the imputation. Ten imputations were undertaken with 5 iterations. Analyses were performed on the imputed datasets and estimates were pooled using the pooled function, which produced a single mean estimate for each parameter with adjusted standard errors according to Rubin’s rules.^34^ Convergence of the imputation procedure was checked visually, with trajectories or streams of the mean and SD of each plotted imputed variable. The plausibility of the imputations was checked by visualizing discrepancies between the observed and imputed data.

#### Deficit Accumulation FI

A complete-case analysis was undertaken for models using the FI to characterize frailty. Participants with missing baseline data for eGFR, UACR, frailty (< 50 FI items), or missing data for frailty at any follow-up visit were excluded from the FI analysis.

### Main Analyses

The main analyses for each of the 2 outcome variables (FP and FI) were the same. A discrete time, complementary log-log regression survival model (equivalent to Cox regression^35^) was used to assess whether baseline eGFR and/or UACR were associated with time to incident frailty, as ascertained by each criterion. The analyses produced HRs and 95% CIs.

Separate analyses were undertaken using 3 different predictor variables: baseline eGFR (categorical), baseline eGFR (continuous), and log_2_-transformed baseline UACR (continuous). For the eGFR analyses, 3 models were estimated, including an unadjusted model with no covariates, an age- and sex-adjusted model (adjusted model 1); and a fully adjusted model (adjusted model 2) including age, sex, log_2_(UACR), body mass index, country of residence, ASPREE treatment group, diabetes, mean systolic and diastolic blood pressure, smoking history, alcohol consumption history, baseline prefrail/not prefrail status, and education. For the UACR analysis, a similar approach was used; eGFR was included as a covariate in the fully adjusted model.

Because we hypothesized that a nonlinear, potentially J-shaped, relationship exists between creatinine based eGFR and incident frailty, we formally tested this with fractional polynomial transformations using the *mfpmi* Stata package for multiple imputed data.^36^^,^^37^ After initial testing, the best fitting functional form for the relationship between eGFR and incident frailty was a second-degree fractional polynomial transformation (*P* < 0.001 compared to the linear model). The second-degree fractional polynomial–transformed eGFR variables were used for all baseline continuous eGFR analyses. Because the HRs for the transformed eGFR variables are themselves not directly interpretable, model results were presented in 2 ways. First, the nonlinear relationship between eGFR and incident frailty was plotted graphically using the model-based coefficients; and second, representative HRs were calculated across the range of eGFR values in the study population, with a reference eGFR of 75 ml/min per 1.73 m^2^.

### Sensitivity Analysis

Two sensitivity analyses were performed. First, a complete case analysis, without imputation of missing data, was undertaken using the FP to characterize frailty. A comparison was made between results from the observed and imputed data analyses. Second, all analyses were repeated using the 2009 CKD-EPI equation (CKD-EPI_2009_)^38^ to calculate eGFR because this is the eGFR equation currently in use Australia and other countries outside North America. Further details are provided in the Supplementary Methods.

This manuscript was prepared according to STROBE cohort guidelines for observational studies.^39^

## Results

### Fried FP

In the first main analysis, the FP was used to measure frailty. There were 19,114 participants enrolled in the ASPREE trial cohort. Of these, 1350 had missing baseline eGFR and/or UACR, and a further 799 were either frail at baseline or had missing baseline frailty data. After all exclusions, 16,965 participants were included for multiple imputation of remaining missing values, and analysis (Supplementary Figure S1).

Baseline demographic characteristics of the included participants, stratified by baseline eGFR category are summarized in Table 1. Overall, 56% of participants were female, with a mean age of 75.0 ± 4.5 years. The median eGFR was 78.6 ml/min per 1.73 m^2^ (IQR: 67.6–89.5) and median UACR was 0.8 mg/mmol (IQR 0.5, 1.5). A histogram demonstrating the overall distribution of UACR at baseline is shown in Supplementary Figure S2. At baseline, 40% of (*n* = 6709) participants were prefrail.

**Table 1:** Baseline characteristics of ASPREE participants overall and by baseline eGFR value using the Fried frailty phenotype to measure frailty

Characteristics	Overall (*N* = 16,965)	Baseline eGFR (ml/min per 1.73 m^2^)			
		< 45 (*n* = 369, 2%)	45–59 (*n* = 1794, 11%)	60–89 (*n* = 10,842, 64%)	≥ 90 (*n* = 3960, 23%)
Age (yrs), mean (SD)	75.0 (4.5)	77.9 (5.7)	76.9 (5.2)	75.4 (4.5)	73.0 (2.9)
Female, *n* (%)	9521 (56%)	229 (62%)	1076 (60%)	5959 (55%)	2257 (57%)
Country, *n* (%)					
Australia	14,783 (87%)	303 (82%)	1472 (82%)	9589 (88%)	3419 (86%)
USA	2182 (13%)	66 (18%)	322 (18%)	1253 (12%)	541 (14%)
Race, n (%)					
White, Australian	14,580 (86%)	297 (81%)	1454 (81%)	9452 (87%)	3377 (85%)
White, US	1126 (6.6%)	18 (4.9%)	132 (7.4%)	701 (6.5%)	275 (6.9%)
Hispanic	218 (1.3%)	3 (0.8%)	15 (0.8%)	102 (0.9%)	98 (2.5%)
African American	802 (4.7%)	43 (12%)	172 (9.6%)	432 (4.0%)	155 (3.9%)
Other	230 (1.4%)	7 (1.9%)	21 (1.2%)	147 (1.4%)	55 (1.4%)
ASPREE treatment group, *n* (%)					
Aspirin	8432 (50%)	198 (54%)	898 (50%)	5385 (50%)	1951 (49%)
Placebo	8533 (50%)	171 (46%)	896 (50%)	5457 (50%)	2009 (51%)
Prefrail^a^, *n* (%)	6709 (40%)	218 (59%)	838 (47%)	4196 (39%)	1457 (37%)
Education (yrs), *n* (%)					
< 12	7601 (49%)	171 (51%)	849 (52%)	4834 (49%)	1747 (49%)
≥12	7790 (51%)	165 (49%)	788 (48%)	5003 (51%)	1834 (51%)
Smoking history, *n* (%)					
Current	648 (3.8%)	12 (3.3%)	67 (3.7%)	376 (3.5%)	193 (4.9%)
Former	6951 (41%)	139 (38%)	720 (40%)	4449 (41%)	1643 (41%)
Never	9366 (55%)	218 (59%)	1007 (56%)	6017 (55%)	2124 (54%)
Alcohol consumption history, *n* (%)					
Current	13,053 (77%)	238 (64%)	1280 (71%)	8394 (77%)	3141 (79%)
Former	1004 (5.9%)	36 (9.8%)	115 (6.4%)	622 (5.7%)	231 (5.8%)
Never	2908 (17%)	95 (26%)	399 (22%)	1826 (17%)	588 (15%)
Body mass index (kg/m^2^), mean (SD)	28.0 (4.7)	29.5 (5.3)	28.7 (4.8)	28.0 (4.5)	27.8 (4.9)
Body mass index, *n* (%)					
< 20	317 (2%)	2 (0.5%)	22 (1%)	177 (2%)	166 (3%)
20–24.9	4166 (25%)	60 (16%)	348 (19%)	2692 (25%)	1066 (27%)
25–29.9	7543 (44%)	167 (45%)	816 (45%)	4921 (45%)	1639 (41%)
30–34.9	3604 (21%)	93 (25%)	438 (24%)	2241 (21%)	832 (21%)
≥ 35	1335 (8%)	47 (13%)	170 (10%)	811 (8%)	307 (8%)
Hypertension, *n* (%)	12,586 (74%)	339 (92%)	1503 (84%)	7997 (74%)	2747 (69%)
Blood pressure (mm Hg), mean (SD)					
Systolic	139 (16)	141 (17)	140 (17)	139 (16)	139 (16)
Diastolic	77 (10)	76 (12)	76 (10)	77 (10)	78 (10)
Diabetes mellitus, *n* (%)	1805 (11%)	67 (18%)	271 (15%)	1049 (9.7%)	418 (11%)
Polypharmacy, *n* (%)	4427 (26%)	190 (51%)	630 (35%)	2691 (25%)	916 (23%)
Dyslipidemia, *n* (%)	11,103 (65%)	263 (71%)	1198 (67%)	7119 (66%)	2523 (64%)
UACR (mg/mmol), median (IQR)	0.8 (0.5–1.5)	1.3 (0.7–3.9)	0.9 (0.5–2.0)	0.8 (0.4–1.4)	0.9 (0.5–1.5)

ASPREE, ASPirin in Reducing Events in the Elderly; CKD-EPI, Chronic Kidney Disease Epidemiology Collaboration; eGFR, estimated glomerular filtration rate (calculated with the 2021 CKD-EPI equation); IQR, interquartile range; SD, standard deviation; UACR, urine albumin-to-creatinine ratio.

Observed data; not imputed.

a Prefailty defined using the Fried frailty phenotype score. A score of 0 indicates no frailty, 1 or 2 is “prefrail” and 3, 4 or 5 is ‘frail.”

More than half of participants (*n* = 10,842, 64%) had a baseline eGFR between 60 and 89 ml/min per 1.73 m^2^, whereas 2% (*n* = 369) had a baseline eGFR < 45 ml/min per 1.73 m^2^. Participants with an eGFR < 45 ml/min per 1.73 m^2^ were more likely to be prefrail at baseline; and have polypharmacy, diabetes, and a higher median UACR. Those with a baseline eGFR ≥ 90 ml/min per 1.73 m^2^ were on average younger, had a lower prevalence hypertension, and were more likely to be currently consuming alcohol.

### Albuminuria and Incident Frailty

**Figure 1:**
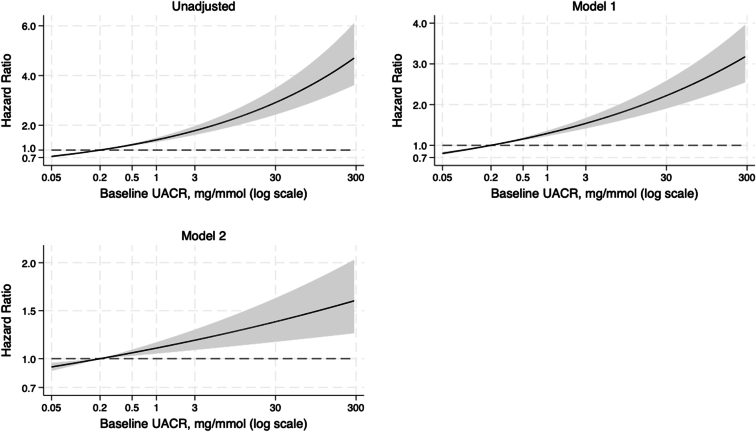
Multivariable-adjusted hazard ratios for incident frailty associated with urine albumin-to-creatinine ratio (log scale). Model 1 adjusted for age and sex; model 2 adjusted for age, sex, estimated glomerular filtration rate, smoking history, alcohol consumption history, body mass index, education, polypharmacy, dyslipidemia, ASPREE study treatment (aspirin or placebo), prefrailty status, country of residence, diabetes, systolic blood pressure, and diastolic blood pressure. Incident frailty was measured using Fried frailty phenotype. ASPREE, ASPirin in Reducing Events in the Elderly.

The median follow-up time was 4.0 years (IQR: 3.0–5.0). Across the 10 imputed data sets, an average of 2429 participants developed incident frailty. Analysis of the relationship between albuminuria and incident frailty is summarized in Table 2 and shown in Figure 1. In all models, there was a significant relationship between increasing albuminuria and the risk of incident frailty. In the fully adjusted model 2, for every doubling of UACR, the risk of incident frailty increased by 4% (HR: 1.04, 95% CI: 1.02–1.07).

**Table 2:** Hazard ratio baseline log_2_-transformed baseline urine albumin-to-creatinine ratio in discrete time survival analysis of incident frailty using 2 measures of frailty

Cox proportional hazards model	Fried frailty phenotype HR (95% CI)^a^	Deficit accumulation frailty index HR (95% CI)
Unadjusted	1.16 (1.13–1.18) *P* < 0.001	1.14 (1.11–1.17) *P* < 0.001
Adjusted 1^b^	1.11 (1.09–1.13) *P* < 0.001	1.11 (1.08–1.14) *P* < 0.001
Adjusted 2^c^	1.04 (1.02–1.07) *P* = 0.002	1.04 (1.01–1.07) *P* = 0.003

CI, confidence interval; HR, hazard ratio.

a Pooled estimates of 10 imputed datasets using Rubin’s rules.

b Model 1, adjusted for age and sex.

c Model 2, adjusted for age, sex, estimated glomerular filtration rate, smoking history, alcohol consumption history, body mass index, education, polypharmacy, dyslipidemia, ASPREE study treatment (aspirin or placebo), prefrailty status, country of residence, diabetes, systolic blood pressure, and diastolic blood pressure.

### eGFR and Incident Frailty

**Figure 2:**
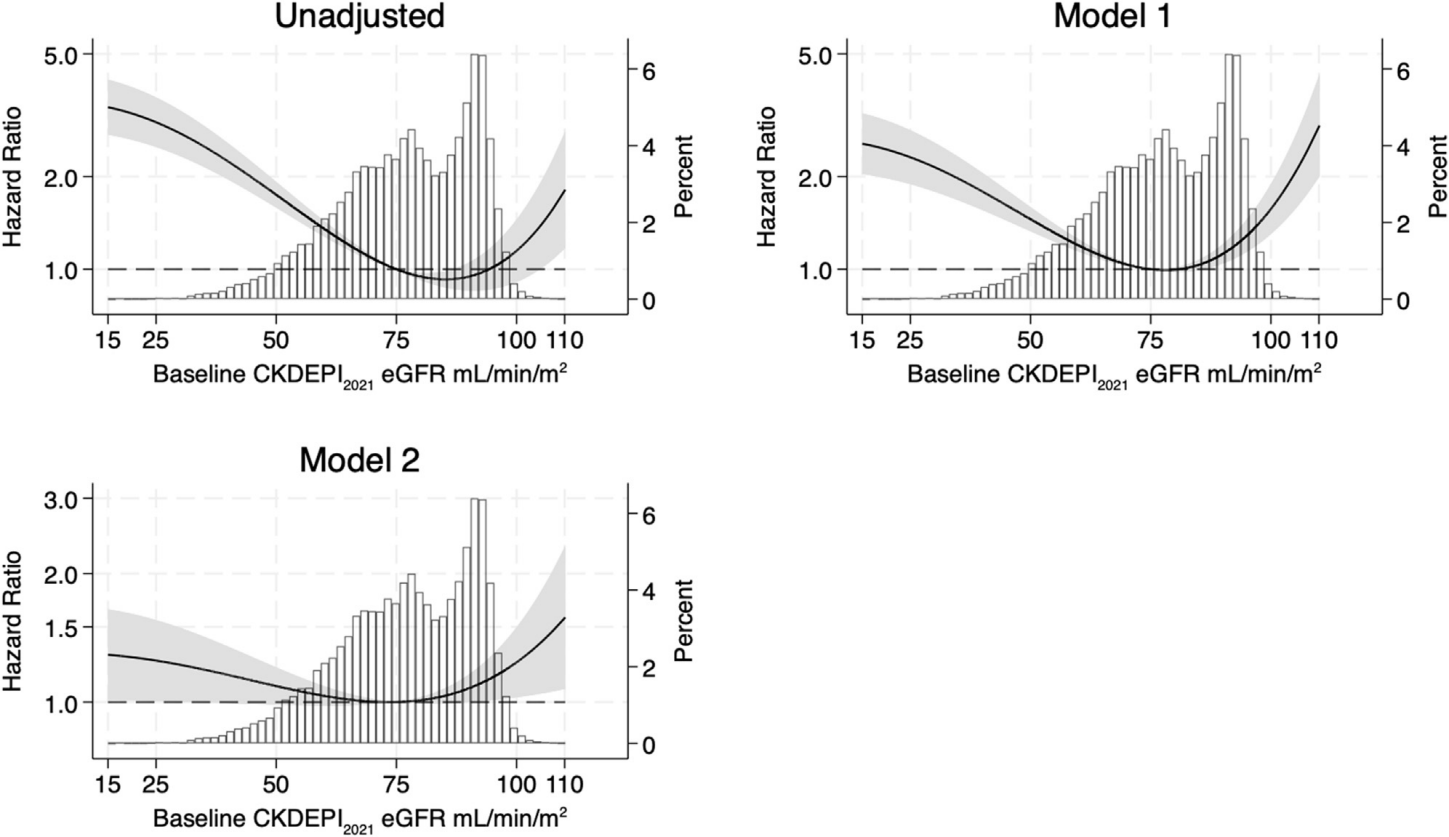
Multivariable-adjusted hazard ratios for incident frailty associated with eGFR (10 imputed datasets) with superimposed histogram of eGFR distribution in the analysis cohort (second y-axis). Values calculated using fractional polynomials for eGFR. Model 1 is adjusted for age and sex. Model 2 is adjusted for age, sex, log2(urine albumin-to-creatinine ratio), smoking history, alcohol consumption history, body mass index, education, polypharmacy, dyslipidemia, ASPREE study treatment (aspirin or placebo), prefrailty status, country of residence, diabetes, systolic blood pressure, and diastolic blood pressure. Powers of fractional polynomials for eGFR 3, 3. Incident frailty was measured using Fried frailty phenotype. ASPREE, ASPirin in Reducing Events in the Elderly; eGFR, estimated glomerular filtration rate.

The association between baseline eGFR and incident frailty in the unadjusted and adjusted models are shown in Figure 2. A J-shaped relationship was observed, and both low and high eGFR levels were significantly associated with an increased risk of incident frailty; this relationship persisted in the fully adjusted model. Representative HRs from the fully adjusted model 2, referenced to eGFR of 75 ml/min per 1.73 m^2^, are shown in Table 3. A reference of 75 ml/min per 1.73 m^2^ was chosen because it was observed that the HR for incident frailty was close to the median eGFR for the population and was associated with the lowest risk of incident frailty. Individuals with an eGFR at baseline that was < 30 ml/min per 1.73 m^2^ or ≥ 95 ml/min per 1.73 m^2^ had an increased risk of incident frailty.

**Table 3:** Representative hazard ratios for incident frailty, measured using the Fried frailty phenotype, as a function of baseline creatinine-based eGFR

Baseline eGFR (ml/min per 1.73 m^2^)	Unadjusted HR (95% CI)^a^	Model 1^b^ HR (95% CI)^a^	Model 2^c^ HR (95% CI)^a^
30	2.76 (2.33–3.28)	2.15 (1.79–2.58)	1.22 (1.00–1.49)
45	1.98 (1.76–2.23)	1.61 (1.44–1.81)	1.12 (0.99–1.27)
60	1.35 (1.28–1.43)	1.19 (1.14–1.81)	1.03 (0.98–1.09)
70	1.09 (1.06–1.11)	1.04 (1.02–1.05)	1.00 (0.99–1.02)
75	1.00 (reference)	1.00 (reference)	1.00 (reference)
80	0.95 (0.92–0.97)	1.00 (0.98–1.01)	1.01 (0.99–1.03)
95	1.01 (0.86–1.19)	1.29 (1.14–1.45)	1.14 (1.01–1.29)
100	1.15 (0.91–1.45)	1.57 (1.30–1.89)	1.24 (1.03–1.50)
110	1.80 (1.17–2.78)	2.90 (1.99–4.24)	1.57 (1.07–2.31)

CI: confidence interval. eGFR: estimated glomerular filtration rate HR: hazard ratio.

a Pooled estimates of 10 imputed datasets using Rubin’s rules.

b Model 1, adjusted for age and sex.

c Model 2, adjusted for age, sex, log2(urine albumin-to-creatinine ratio), smoking history, alcohol consumption history, body mass index, education, polypharmacy, dyslipidemia, ASPREE study treatment (aspirin or placebo), prefrailty status, country of residence, diabetes, systolic blood pressure, and diastolic blood pressure.

Further analyses with baseline eGFR divided into 4 categories (eGFR <45, 45–59, 60–89, ≥90 ml/min per 1.73 m^2^) are presented in Supplementary Table S3. There was no difference in the risk of incident frailty between the eGFR categories in the fully adjusted model.

### Sensitivity Analysis

Analyses were repeated using the original observed data (without imputation) to assess the risk of incident frailty. Overall, 15,391 participants were included in this analysis (Supplementary Figure S1). Over a median of 4.9 years (IQR: 3.9–5.1), 950 people (6%) developed frailty. Results of the albuminuria analysis were similar to the main analysis presented in Table 2. The risk of incident frailty increased by 5% (HR: 1.05, 95% CI: 1.01–1.09, Supplementary Table S4) per doubling of UACR in the complete case analysis. Results were largely comparable between the imputed data analyses and complete case analyses for the continuous eGFR models (Supplementary Figure S3) and the categorical eGFR models (Supplementary Table S5).

Analyses were repeated using CKD-EPI_2009_ to calculate eGFR, and the results are shown in Supplementary Tables S6 to S10 and Supplementary Figures S4 and S5. The results were largely consistent with results from analyses using CKD-EPI_2021_. Imputed data and complete case analysis for the eGFR categorical model and the FP measure of frailty showed that, compared with participants with a baseline eGFR between 60 and 89 ml/min per 1.73 m^2^, participants with a baseline eGFR ≥ 90 ml/min per 1.73 m^2^ had an increased risk of incident frailty in fully adjusted models.

### Deficit Accumulation FI

In the second main analysis, the FI was used as an alternative measure of frailty. Overall, 5855 participants had missing baseline eGFR, UACR, and/or data for confounders, and a further 987 were either frail at baseline, or had missing baseline or longitudinal frailty data. After excluding these, 12,272 participants were included for analysis (Supplementary Figure S6).

Baseline demographic characteristics of the included participants, stratified by baseline eGFR category are summarized in Table 4. Overall, 54% of participants were female, with a mean age of 74.8 ± 4.3 years. The median eGFR was 79.2 ml/min per 1.73 m^2^ (IQR: 68.2–89.7) and median UACR was 0.8 mg/mmol (IQR: 0.5–1.4). At baseline, 44% (*n* = 5438) of participants were prefrail.

**Table 4:** Baseline characteristics of ASPREE participants included for analyses using the deficit accumulation frailty index to measure frailty

Characteristics	Overall (*N* = 12,272)	Baseline eGFR (ml/min per 1.73 m^2^)			
		< 45 (*n* = 215, 2%)	45–59 (*n* = 1174, 9%)	60–89 (*n* = 7930, 65%)	≥ 90 (*n* = 2953, 24%)
Age (yrs), mean (SD)	74.8 (4.3)	77.6 (5.5)	76.6 (5.0)	75.2 (4.3)	73.0 (2.8)
Female, *n* (%)	6607 (54%)	128 (60%)	649 (55%)	4195 (53%)	1635 (55%)
Country, *n* (%)					
Australia	11,037 (90%)	183 (85%)	1007 (86%)	7198 (91%)	2649 (90%)
USA	1235 (10%)	32 (15%)	167 (14%)	732 (9.2%)	304 (10%)
Race, n (%)					
White, Australian	10,901 (89%)	179 (83%)	996 (85%)	7108 (90%)	2618 (89%)
White, US	674 (5.5%)	10 (4.7%)	75 (6.4%)	432 (5.5%)	157 (5.3%)
Hispanic	133 (1.1%)	2 (0.9%)	7 (0.6%)	67 (0.8%)	57 (1.9%)
African American	414 (3.4%)	20 (9.3%)	84 (7.2%)	228 (2.9%)	82 (2.8%)
Other	145 (1.2%)	4 (1.9%)	12 (1.0%)	90 (1.1%)	39 (1.3%)
ASPREE treatment group, *n* (%)					
Aspirin	6066 (49%)	119 (55%)	569 (48%)	3910 (49%)	1468 (50%)
Placebo	6206 (51%)	96 (45%)	605 (52%)	4020 (51%)	1485 (50%)
Prefrail^a^, *n* (%)	5438 (44%)	159 (74%)	761 (65%)	3323 (42%)	1195 (40%)
Education (years), *n* (%)					
< 12	5940 (48%)	105 (49%)	591 (50%)	3824 (48%)	1420 (48%)
≥ 12	6332 (52%)	110 (51%)	583 (50%)	4106 (52%)	1533 (52%)
Smoking history, *n* (%)					
Current	412 (3.4%)	4 (1.9%)	37 (3.2%)	240 (3.0%)	131 (4.4%)
Former	4950 (40%)	83 (39%)	463 (39%)	3191 (40%)	1213 (41%)
Never	6910 (56%)	128 (60%)	674 (57%)	4499 (57%)	1609 (54%)
Alcohol consumption history, *n* (%)					
Current	9618 (78%)	141 (66%)	873 (74%)	6233 (79%)	2371 (80%)
Former	644 (5.2%)	14 (6.5%)	58 (4.9%)	408 (5.1%)	164 (5.6%)
Never	2010 (16%)	60 (28%)	243 (21%)	1289 (16%)	418 (14%)
Body mass index (kg/m^2^), mean (SD)	27.8 (4.4)	29.0 (5.0)	28.3 (4.4)	27.8 (4.3)	27.6 (4.7)
Body mass index, *n* (%)					
< 20	209 (1.7%)	3 (1.4%)	15 (1.3%)	110 (1.4%)	81 (2.7%)
20–24.9	3078 (25%)	35 (16%)	240 (20%)	1981 (25%)	822 (28%)
25–29.9	5691 (46%)	102 (47%)	568 (48%)	3767 (48%)	1254 (42%)
30–34.9	2470 (20%)	47 (22%)	271 (23%)	1554 (20%)	598 (20%)
≥ 35	824 (6.7%)	28 (13%)	80 (6.8%)	518 (6.5%)	198 (6.7%)
Hypertension, *n* (%)	8982 (73%)	196 (91%)	949 (81%)	5789 (73%)	2048 (69%)
Blood pressure (mm Hg), mean (SD)					
Systolic	139 (16)	140 (17)	140 (17)	139 (16)	139 (16)
Diastolic	77 (10)	76 (11)	76 (10)	77 (10)	77 (10)
Diabetes mellitus, *n* (%)	1186 (9.7%)	35 (16%)	154 (13%)	695 (8.8%)	302 (10%)
Polypharmacy, *n* (%)	2777 (23%)	95 (44%)	334 (28%)	1701 (21%)	647 (22%)
Dyslipidemia, *n* (%)	8022 (65%)	142 (66%)	774 (66%)	5203 (66%)	1903 (64%)
UACR (mg/mmol), median (IQR)	0.80 (0.50–1.40)	1.20 (0.60–3.30)	0.90 (0.50–1.70)	0.70 (0.40–1.30)	0.80 (0.50–1.40)

ASPREE, ASPirin in Reducing Events in the Elderly; eGFR, estimated glomerular filtration rate; IQR, interquartile range; SD, standard deviation; UACR, urine albumin to creatinine ratio.

Baseline demographic variables of participants who were not frail at the time of entry to the ASPREE trial.

Observed data; not imputed.

a Prefrail defined as a frailty index score of greater than 0.10 and less than or equal to 0.21.

### Albuminuria and Incident Frailty

**Figure 3:**
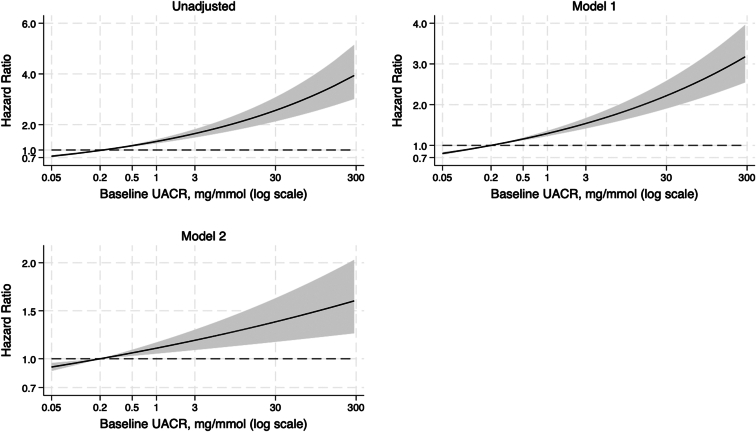
Multivariable-adjusted hazard ratios for incident frailty associated with urine albumin-to-creatinine ratio (log scale). Model 1 adjusted for age and sex; model 2 adjusted for age, sex, estimated glomerular filtration rate, smoking history, alcohol consumption history, body mass index, education, polypharmacy, dyslipidemia, ASPREE study treatment (aspirin or placebo), prefrailty status, country of residence, diabetes, systolic blood pressure, and diastolic blood pressure. Incident frailty was measured using the deficit accumulation frailty index. ASPREE, ASPirin in Reducing Events in the Elderly.

The risk of incident FI frailty, increased with increasing baseline UACR, consistent with the FP frailty outcome. HRs for the unadjusted model and adjusted models 1 and 2 are summarized in Table 2 and shown in Figure 3. For every doubling of baseline UACR, the risk of incident FI frailty increased by 4% (HR:1.04, 95% CI:1.01–1.07) in the adjusted model 2.

### eGFR and Incident Frailty

**Figure 4:**
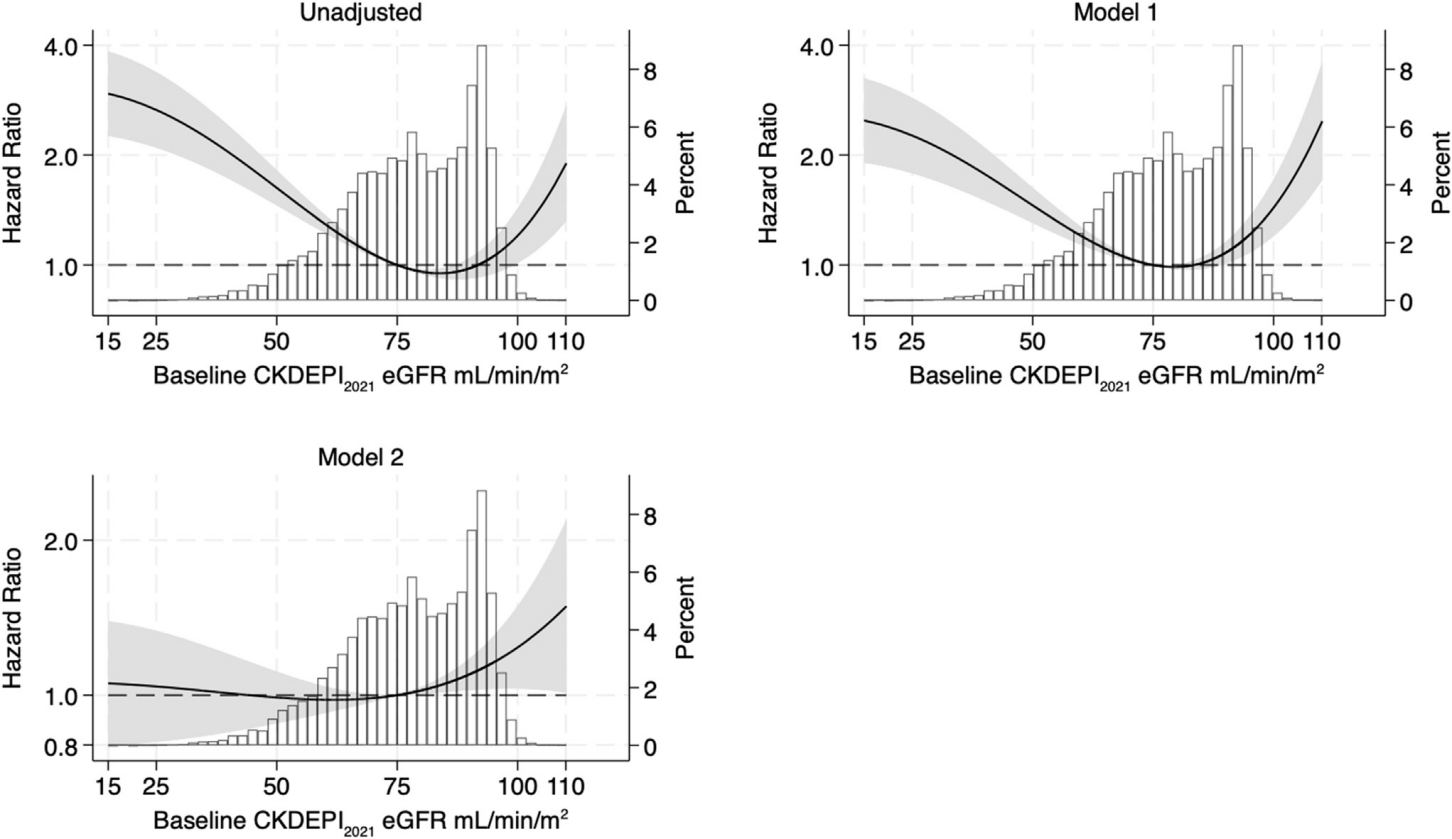
Hazard ratios for incident frailty associated with eGFR with superimposed histogram of eGFR distribution in the observed data (second y-axis). Values calculated using fractional polynomials for eGFR. Top left unadjusted model, top right: model 1, age- and sex-adjusted; bottom left: model 2 fully adjusted. Powers of fractional polynomials for eGFR 3, 3. Incident frailty measured using the deficit accumulation frailty index. ASPREE, ASPirin in Reducing Events in the Elderly; eGFR, estimated glomerular filtration rate.

During a median follow-up period of 4.0 years (IQR: 3.0–5.0), 2338 participants developed frailty according to the FI. The estimated associations between the risk of incident frailty and baseline eGFR were largely consistent with the findings of the FP outcome in both the categorical and the continuous eGFR models (Figure 4 and Supplementary Table S3).

## Discussion

We examined the association between baseline eGFR and UACR with the risk of incident frailty, as ascertained by 2 separate frailty scales, in a large cohort of relatively healthy, older, and community-dwelling adults. There were 2 main findings in this study. First, albuminuria was associated with an increased risk of incident frailty after adjusting for potential confounders, including eGFR. Second, a nonlinear J-shaped relationship was observed between baseline kidney function (i.e., eGFR) and the risk of incident frailty. Findings remained consistent whether using the FI or the FP to determine incident frailty.

We identified that a doubling of baseline UACR was associated with incident frailty, independent of eGFR, even though the median overall UACR was well within the normal limits. These findings are similar to a study by Chang *et al.*,^25^ who found that even very low levels of albuminuria predicted incident prefrailty and frailty in 1441 middle-aged and older adults from Taiwan. In addition, Bůžková *et al.*^10^ identified that a 2-fold increase in UACR was associated with decline in grip-strength and slower gait speed in >2000 older adults over 2 years follow-up. In our study, we provide valuable insights into the association between very low-level urine albumin and incident frailty, given that ASPREE is a large cohort of relatively healthy older individuals with a longer follow-up period.

The mechanism underlying the association between albuminuria and incident frailty is uncertain. Albuminuria is linked to systemic inflammation, with past studies identifying increased plasma levels of proinflammatory cytokines and acute phase reactants in individuals with higher levels of urinary albumin.^40^ Inflammation has also been directly implicated in the pathogenesis of frailty. The term ”inflammageing” describes a state of accelerated ageing, a process mediated by inflammation and predispose to frailty.^41^ The current Kidney Disease Improving Global Outcomes definition of CKD accepts urine albumin levels < 3 mg/mmol as within the normal range; however, as we have shown, even small increases of urine albumin below this cut-off, were associated with increased risk of incident frailty.^42^ Routine testing of urine albumin is not currently recommended in older patients without specific indication, and it is unclear whether agents known to target albuminuria, such as angiotensin receptor blockers or angiotensin-converting enzyme inhibitors, have a role in reducing incident frailty in older patients.^43^ Albuminuria may occur transiently in the setting of acute inflammatory states or intense physical activity, and must therefore be repeated in clinical practice to confirm persistence.^42^ In our study, analyses were based on a single measure of UACR. Nonetheless, detection of albuminuria in older patients without significant comorbidities may help identify those at increased risk for frailty and assist in early intervention and prevention.

We identified a nonlinear association between baseline eGFR and incident frailty, with increased incidence of frailty in participants with both high and low eGFR. In categorical analyses, the association between eGFR ≥ 90 ml/min per 1.73 m^2^ and incident frailty was statistically significant when using CKD-EPI_2009_, but not CKD-EPI_2021_, to calculate eGFR. On average, CKD-EPI_2021_ estimates a higher GFR than CKD-EPI_2009_,^44^ which would have shifted a proportion of people who would otherwise have borderline eGFR (just <90 ml/min per 1.73 m^2^) into the ≥ 90 ml/min per 1.73 m^2^ eGFR category. Given that ASPREE is a cohort of generally healthy older individuals, it is likely that the individuals who were recategorized from having an eGFR of slightly < 90 ml/min per 1.73 m^2^ using CKD-EPI_2009_, to having an eGFR ≥ 90 ml/min per 1.73 m^2^ using CKD-EPI_2021_, were healthy and less likely to develop incident frailty. For high eGFR, the results of the eGFR analyses are consistent with findings in a US cohort of >4000 adults aged ≥65 years, where serum creatinine–based but not cystatin C–based eGFR ≥ 90 ml/min per 1.73 m^2^ was associated with a higher risk of incident frailty.^22^ In contrast, Guerville *et al.*^45^ found no association between baseline eGFR and incident frailty over 3 to 5 years of follow-up in >2000 community-dwelling adults aged ≥70 years with memory complaints and/or dependent on assistance for at least 1 activity of daily living. However, eGFR was dichotomized (< or ≥ 60 ml/min per 1.73 m^2^), which may have masked an association between higher eGFR (≥ 90 ml/min per 1.73 m^2^) and frailty. Although there is a paucity of quality studies investigating the relationship between elevated eGFR and incident frailty, the association between elevated creatinine-based eGFR and negative health outcomes in older adults has been described elsewhere.^46^^,^^47^ Elevated baseline eGFR > 105 ml/min per 1.73 m^2^ predicted a 5-fold increased risk, relative to eGFR of 60 to 74.9 ml/min per 1.73 m^2^, for all-cause mortality in Canadian adults aged > 60 years in a prospective cohort study; frailty was not assessed.^15^

Potential mechanisms linking increased serum creatinine–based eGFR and incident frailty must be considered. Muscle wasting, weakness, or severe chronic disease can lead to lower serum creatinine, and therefore, increased eGFR, despite no change in kidney function.^28^ Cystatin C is less influenced by muscle mass and high eGFR calculated with cystatin C is not associated with prevalent frailty^22^^,^^48^ whereas individuals with a higher eGFR estimated by serum creatinine compared to cystatin C were more likely to be frail.^49^ The association between elevated creatinine-based eGFR and incident frailty is therefore likely a function of sarcopenia, rather than true preservation in kidney function. Body composition is challenging to accurately measure in clinical practice, and body mass index is an unreliable indicator of muscle mass and/or total body fat, particularly in older adults.^50^ Therefore, although our analysis adjusted for body mass index, it may not have fully accounted for changes in body composition, including sarcopenia with or without abdominal adiposity.

For those individuals with reduced eGFR < 45 ml/min per 1.73 m^2^, our results are consistent with a previous smaller study.^3^ In that cohort of 669 community-dwelling older adults, those with eGFR < 45 ml/min per 1.73 m^2^ had a 2.5-fold higher risk of incident frailty compared to those with eGFR ≥ 60 ml/min per 1.73 m^2,3^ Although the results were similar, participants in that cohort were older (mean age 84 years) and had significant comorbid diseases. In addition, the analysis did not adjust for baseline UACR, polypharmacy or dyslipidemia and a substantial number of participants were excluded because of missing data. Frailty is common in people with moderate to advanced CKD.^51^, ^52^, ^53^ These conditions are frequently comorbid because they share common risk factors, which are particularly common in older age. Visceral obesity, cellular senescence, impaired elimination of degraded cellular material, and atherosclerosis contribute to chronic inflammation, which is a risk factor for CKD.^41^ In addition, individuals with CKD often have loss of appetite and reduced energy intake which contributes to muscle wasting, reduced physical activity, and frailty.^51^ Other factors, including cognitive impairment, depression, uremia, metabolic acidosis, hyperparathyroidism, and polypharmacy are common in people with advanced CKD and contribute to the frailty syndrome.^51^^,^^54^^,^^55^

Our study has several strengths. ASPREE was a large, binational, longitudinal primary prevention study; and thus, our results should generalize to healthy older adults in Australia and the USA. Our findings were strengthened using 2 separate complementary measures of frailty. In contrast to previous studies, which excluded participants with missing longitudinal frailty data^3^^,^^22^^,^^45^ and thus may have incurred bias,^56^^,^^57^ we managed missing longitudinal frailty data with multiple imputation.

There were limitations to our study. It would be inappropriate to extend findings from this study to older adults with dementia or an independence-limiting disability. There were substantial missing annual follow-up data in the FP frailty outcome variable because the ASPREE protocol permitted the collection of relevant FP data items at alternate annual visits. To address this, the components of the frailty variable were imputed to enable participants to be included where they had some, but not all, components assessed at a given visit. Concern often arises when an outcome variable is imputed using predictors also used in subsequent final analyses, because imputed values may simply confirm the final model. However, provided a correct statistical model is chosen for analysis and used in both the imputation and final analyses, imputation of an outcome variable does not introduce bias.^58^ There is extensive evidence that many individuals transition between states of frailty, prefrailty and nonfrail.^59^ We assessed time to first frailty only, and multiple imputation may not have appropriately captured the nuance of frailty as a dynamic state, which represents a limitation of our analyses. There was also substantial missing data in the FI index as an outcome variable, and a large proportion of participants with missing data were excluded from that analysis, which may be a source of bias. The results using the FP with either a complete case approach or multiple imputation, and results using the FI, were all consistent, which provides reassurance on the robustness of our overall findings. This study did not examine the relationship between rate of change in eGFR and incident frailty. This has been shown previously as being a predictor of incident frailty.^3^^,^^22^ It is a limitation of this study that our analyses were based on single baseline and annual measurements of serum creatinine, and there was no central laboratory to minimize the risk for variations in assays. Finally, cystatin C was not measured in ASPREE; therefore, it is not possible to compare cystatin C–based eGFR to serum creatinine–based eGFR and their associations with incident frailty.

With early intervention, frailty may be preventable and/or reversible, or at least delayed. This study examined the association between baseline measures of kidney function and long-term risk of incident frailty and found that higher UACR, low eGFR (< 30 ml/min per 1.73 m^2^) and elevated eGFR ≥ 95 ml/min per 1.73 m^2^ are all associated with increased risk of incident frailty in older adults. Older patients with an eGFR < 30 ml or ≥ 95 ml/min per 1.73 m^2^, or albuminuria (even at low levels) should be screened for frailty so that early intervention may be considered.

## Disclosure

All the authors declared no competing interests.

## Supplementary Material

Supplementary File (PDF) **Supplementary Methods. Supplementary References. Figure S1.** Flowchart for participants included for analyses using frailty phenotype. **Figure S2**. Histogram of baseline urine albumin to creatinine ratio. **Figure S3.** Hazard ratios for incident frailty according to the frailty phenotype associated with eGFR (2021 CKD-EPI) in the observed data. **Figure S4.** Pooled hazard ratios of multiple imputed analyses for the association between baseline eGFR (2009 CKD-EPI) and for incident frailty (frailty phenotype). **Figure S5**. Hazard ratios for the association between baseline eGFR (2009 CKD-EPI) and for incident frailty (deficit accumulation frailty index). **Figure S6.** Flowchart for participants included for analyses using the frailty index. **Table S1.** Missing data for each of the frailty phenotype components at each follow-up visit. **Table S2.** Imputation methods. **Table S3.** Hazard ratios describing the association between baseline estimated glomerular filtration rate (2021 CKD-EPI) and risk of incident frailty. **Table S4.** Hazard ratios for the association between baseline log2-transformed urine albumin-to-creatinine ratio and incident using the Fried frailty phenotype with observed, nonimputed data. **Table S5.** Hazard ratios for baseline estimated glomerular filtration rate (CKD-EPI 2021) in discrete time survival analysis of incident frailty, using the Fried frailty phenotype and observed, non-imputed data. **Table S6.** Baseline characteristics of ASPREE participants overall and by baseline eGFR calculated with 2009 CKD-EPI value using the Fried frailty phenotype to measure frailty. **Table S7.** Baseline characteristics of ASPREE participants included for analyses using the deficit accumulation frailty index to measure frailty, and the 2009 CKD-EPI equation for eGFR. **Table S8.** Hazard ratio for the association between baseline log2-transformed baseline urine albumin-to-creatinine ratio and incident frailty using 2 measures of frailty. The 2009 CKD-EPI equation was used to calculate eGFR and classify CKD for use in the frailty index. **Table S9.** Hazard ratios describing the association between baseline eGFR using 2009 CKD-EPI and risk of incident frailty. **Table S10.** Hazard ratios describing the association between baseline eGFR using 2009 CKD-EPI and incident frailty, using the Fried frailty phenotype and observed, non-imputed data. **Strobe Checklist.**

## Footnotes

Supplementary File (PDF)

**Supplementary Methods.**

**Supplementary References.**
**Figure S1**. Flowchart for participants included for analyses using frailty phenotype. **Figure S2.** Histogram of baseline urine albumin to creatinine ratio. **Figure S3.** Hazard ratios for incident frailty according to the frailty phenotype associated with eGFR (2021 CKD-EPI) in the observed data. **Figure S4.** Hazard ratios from multiple imputation for the association between baseline eGFR (2009 CKD-EPI) and for incident frailty (frailty phenotype). **Figure S5.** Hazard ratios for the association between baseline eGFR (2009 CKD-EPI) and for incident frailty (deficit accumulation frailty index). **Figure S6.** Flowchart for participants included for analyses using the frailty index. **Table S1.** Missing data for each of the frailty phenotype components at each follow-up visit. **Table S2.** Imputation methods. **Table S3.** Hazard ratios describing the association between baseline estimated glomerular filtration rate (2021 CKD-EPI) and risk of incident frailty. **Table S4.** Hazard ratios for the association between baseline log2-transformed urine albumin-to-creatinine ratio and incident using the Fried frailty phenotype with observed, nonimputed data. **Table S5.** Hazard ratios for baseline estimated glomerular filtration rate (CKD-EPI 2021) in discrete time survival analysis of incident frailty, using the Fried frailty phenotype and observed, non-imputed data. **Table S6.** Baseline characteristics of ASPREE participants overall and by baseline eGFR calculated with 2009 CKD-EPI value using the Fried frailty phenotype to measure frailty. **Table S7.** Baseline characteristics of ASPREE participants included for analyses using the deficit accumulation frailty index to measure frailty, and the 2009 CKD-EPI equation for eGFR. **Table S8.** Hazard ratio for the association between baseline log2-transformed baseline urine albumin-to-creatinine ratio and incident frailty using 2 measures of frailty. The 2009 CKD-EPI equation was used to calculate eGFR and classify CKD for use in the frailty index. **Table S9.** Hazard ratios describing the association between baseline eGFR using 2009 CKD-EPI and risk of incident frailty. **Table S10.** Hazard ratios describing the association between baseline eGFR using 2009 CKD-EPI and incident frailty, using the Fried frailty phenotype and observed, non-imputed data.
**Strobe Checklist.**
