# Supplementary material for: Association Between Albuminuria and Glomerular Filtration Rate With Incident Frailty
Source: Kidney Int Rep. 2024 Nov 18;10(2):489–502. doi: 10.1016/j.ekir.2024.11.017 (PMC11843128; doi:10.1016/j.ekir.2024.11.017)
Supplement: Supplementary File (PDF) — Supplementary Methods. Supplementary References. Figure S1. Flowchart for participants included for analyses using frailty phenotype. Figure S2. Histogram of baseline urine albumin to creatinine ratio. Figure S3. Hazard ratios for incident frailty according to the frailty phenotype associated with eGFR (2021 CKD-EPI) in the observed data. Figure S4. Pooled hazard ratios of multiple imputed analyses for the association between baseline eGFR (2009 CKD-EPI) and for incident frailty (frailty phenotype). Figure S5. Hazard ratios for the association between baseline eGFR (2009 CKD-EPI) and for incident frailty (deficit accumulation frailty index). Figure S6. Flowchart for participants included for analyses using the frailty index. Table S1. Missing data for each of the frailty phenotype components at each follow-up visit. Table S2. Imputation methods. Table S3. Hazard ratios describing the association between baseline estimated glomerular filtration rate (2021 CKD-EPI) and risk of incident frailty. Table S4. Hazard ratios for the association between baseline log2-transformed urine albumin-to-creatinine ratio and incident using the Fried frailty phenotype with observed, nonimputed data. Table S5. Hazard ratios for baseline estimated glomerular filtration rate (CKD-EPI 2021) in discrete time survival analysis of incident frailty, using the Fried frailty phenotype and observed, non-imputed data. Table S6. Baseline characteristics of ASPREE participants overall and by baseline eGFR calculated with 2009 CKD-EPI value using the Fried frailty phenotype to measure frailty. Table S7. Baseline characteristics of ASPREE participants included for analyses using the deficit accumulation frailty index to measure frailty, and the 2009 CKD-EPI equation for eGFR. Table S8. Hazard ratio for the association between baseline log2-transformed baseline urine albumin-to-creatinine ratio and incident frailty using 2 measures of frailty. The 2009 CKD-EPI equation was used to calculate eGFR and cl [file mmc1.pdf]

**Supplementary Methods**

*Exposure variables*

The 2021 CKD-EPI equation was used to calculate eGFR in the primary analyses, as it recommended for use by the National Kidney Foundation and the American Society of Nephrology.[S1] At present in Australia and other countries outside North America, kidney function is routinely assessed using the 2009 CKD-EPI equation without using the racial coefficient. As the majority of the ASPREE cohort were resident in Australia (87%), the 2009 CKD-EPI was used in sensitivity analyses to reflect current practice.

A small sample of participants (n=1,348) had a baseline UACR reported in a range (<0.11, 0.11 to 3.38, 3.39 to 33.90, >33.90 mg/mmol) rather than an exact UACR value. For these participants, UACR was manually imputed as 0.11, 2.99, 10.7 and 34 mg/mmol, respectively, as in previous analyses of ASPREE data.[S2,S3]

*Fried Frailty Phenotype*

The Fried frailty phenotype defines five components: unintentional weight loss, slowness, exhaustion, weakness, and low activity.[S4] The score was derived from ASPREE as follows:

- Exhaustion was derived from answers to two Center for Epidemiological Studies-Depression 10 Item Scale (CES-D 10) questions: “I felt that everything I did was an effort” and “I could not get going”. Participants answered based on how they felt the preceding week: rarely or none of the time (less than 1 day), some or a little of the time (1-2 days), occasionally or a moderate amount of time (3-4 days), or all the time (5-7 days). A score of >2 on either question was classified as having exhaustion.
- Weight loss was defined as BMI <20 kg/m$^2$ at baseline or more than 5% weight loss since previous study visit weight.
- Low activity was defined as either no walking outside in the preceding two weeks or walking outside but for less than 10 minutes. On the Life Disability Form, participants would score one point for this component of the Fried phenotype if they answered ‘yes’ to “In the past two weeks, have you done any walking outside the home?”, or if they responded ‘less than 10 minutes’ to “When you walked in the past two weeks, what is the longest amount of time that you walked without sitting down to rest?”
- Weakness was derived from hand grip tests. The mean of three grips from an annual study visit was calculated. Participants with a mean hand grip strength below the 20$^{th}$ percentile for their BMI and sex were classified as having ‘weakness’.
- Slowness was derived from the results of three-meter gait speed tests, measured in seconds. Participants above the 80$^{th}$ percentile for their height and sex for gait speed were classified as having ‘slowness.’

As an additional measure, we assessed incident frailty using the deficit accumulation frailty index (FI), which is a measure of the cumulative sum of health deficits across multiple domains. The FI has been previously validated in ASPREE and described elsewhere.[S5] In short, FI has been adapted to ASPREE as a 67-item measure. The total score for each participant was divided by the total number of items from their frailty assessment with

complete data. An FI score of >0.21 indicates frailty, >0.10 to ≤0.21 indicates prefrailty, and ≤0.10 is non-frail. Only participants with complete data for ≥50 items on the score at every follow-up visit were included for analysis, as the FI was validated in ASPREE as requiring a minimum of 50 items for calculation.

**Supplementary Figure S1.** Derivation of cohort for incident frailty defined using the Fried Frailty phenotype. Participants who were frail at baseline, or if they had missing frailty data at baseline, were excluded from analysis. Participants were also excluded if they were missing baseline kidney function measures (eGFR or UACR).

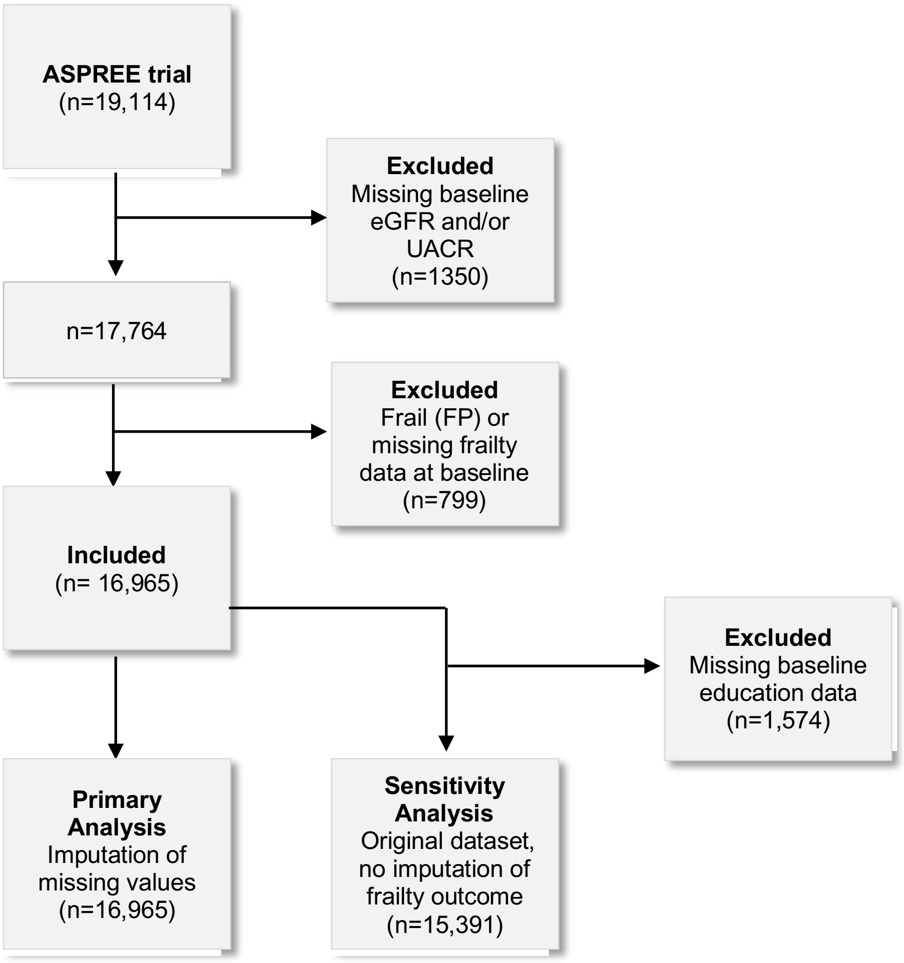


eGFR: estimated glomerular filtration rate, UACR: urine albumin to creatinine ratio; FP: Fried frailty phenotype.

**Supplementary Figure S2:** Histogram of baseline urine albumin to creatinine ratio for all participants included for analysis of incident frailty using the Fried phenotype. UACR: urine albumin to creatinine ratio.

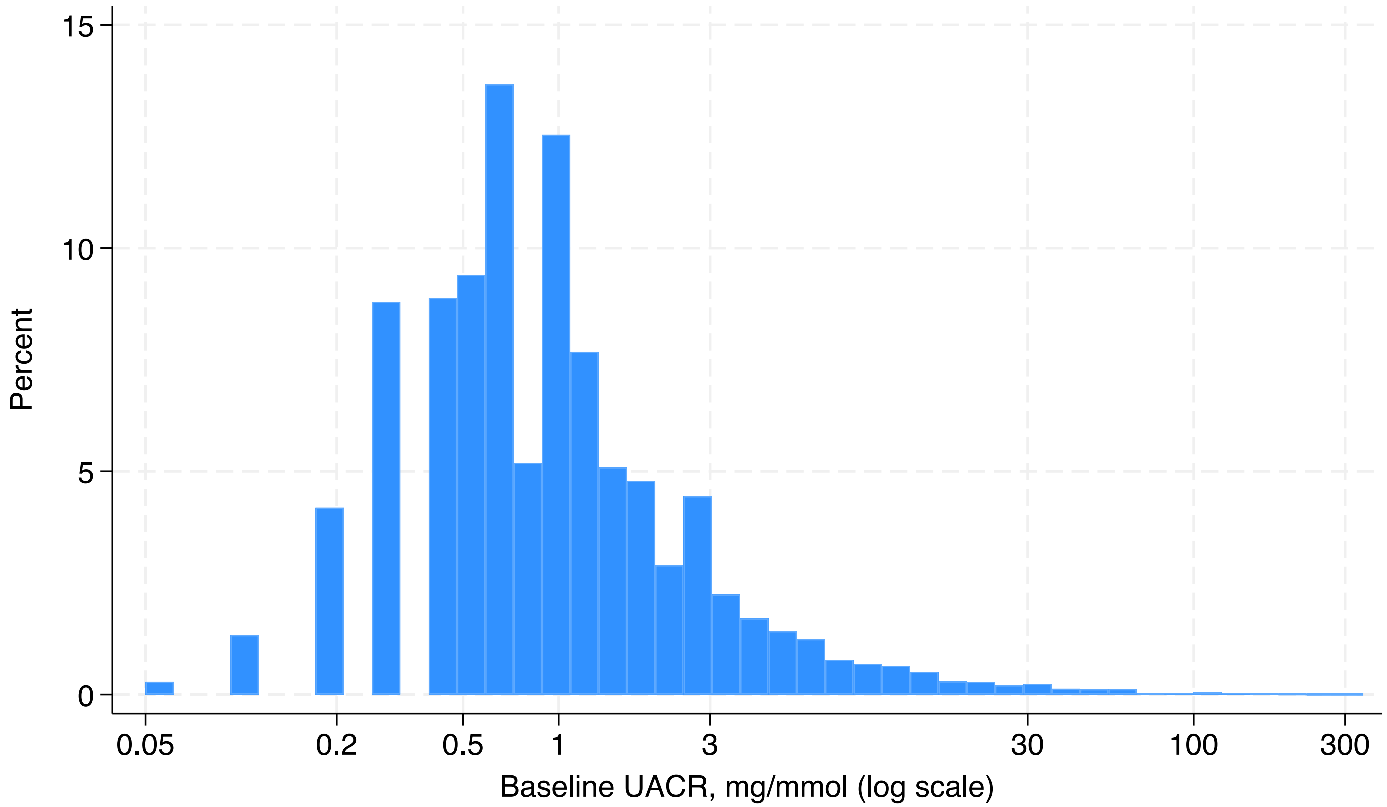

**Supplementary Figure S3:** Hazard ratios for incident frailty associated with eGFR (calculated with 2021 CKD-EPI equation) with superimposed histogram of eGFR distribution in the observed data. Values calculated using fractional polynomials for eGFR. Top left unadjusted model, top right model 1 age and sex adjusted, bottom left model 2 fully adjusted. Powers of fractional polynomials for eGFR 3, 3. Incident frailty was measured using Fried frailty phenotype.

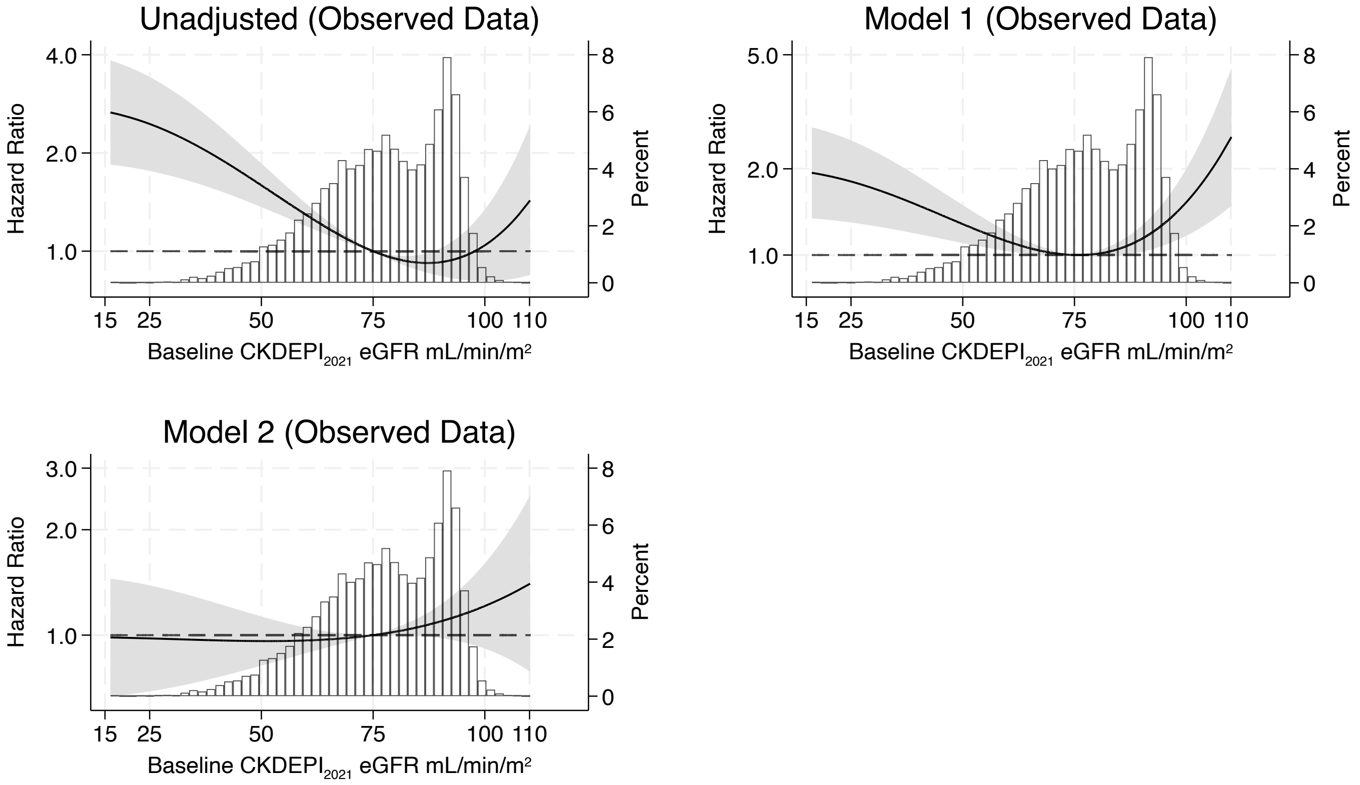


eGFR: estimated glomerular filtration rate; CKD-EPI: Chronic Kidney Disease Epidemiology Collaboration.

**Supplementary Figure S4:** Hazard ratios for incident frailty associated with eGFR (calculated with 2009 CKD-EPI equation) with superimposed histogram of eGFR distribution. Values calculated using fractional polynomials for eGFR. Top left unadjusted model, top right model 1 age and sex adjusted, bottom left model 2 fully adjusted. Powers of fractional polynomials for eGFR 3, 3. Incident frailty was measured using Fried frailty and missing data was managed using multiple imputation.

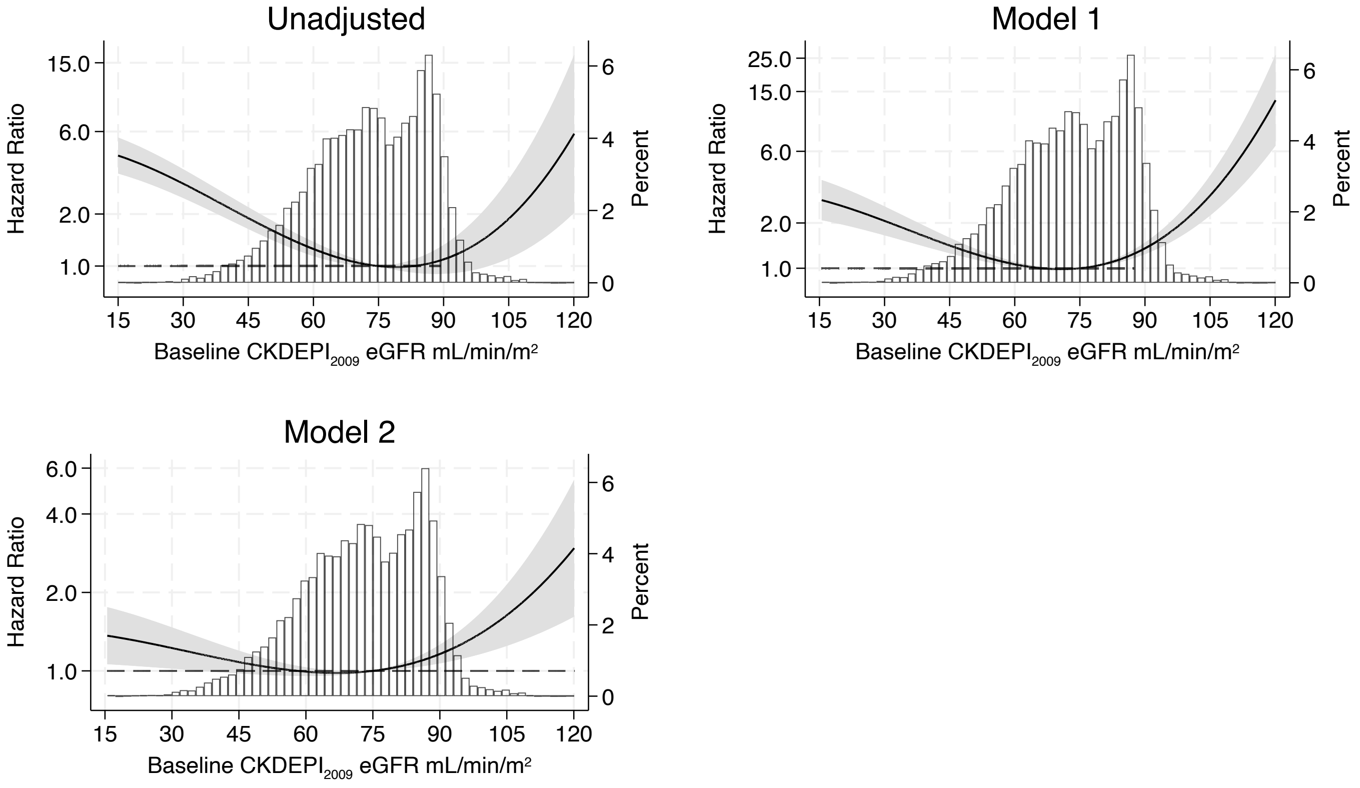


eGFR: estimated glomerular filtration rate; CKD-EPI: Chronic Kidney Disease Epidemiology Collaboration.

**Supplementary Figure S5:** Hazard ratios for incident frailty associated with eGFR (calculated with 2009 CKD-EPI equation) with superimposed histogram of eGFR distribution in the observed data. Values calculated using fractional polynomials for eGFR. Top left unadjusted model, top right model 1 age and sex adjusted, bottom left model 2 fully adjusted. Powers of fractional polynomials for eGFR 3, 3. Incident frailty was measured using the Deficit Accumulation Frailty Index.

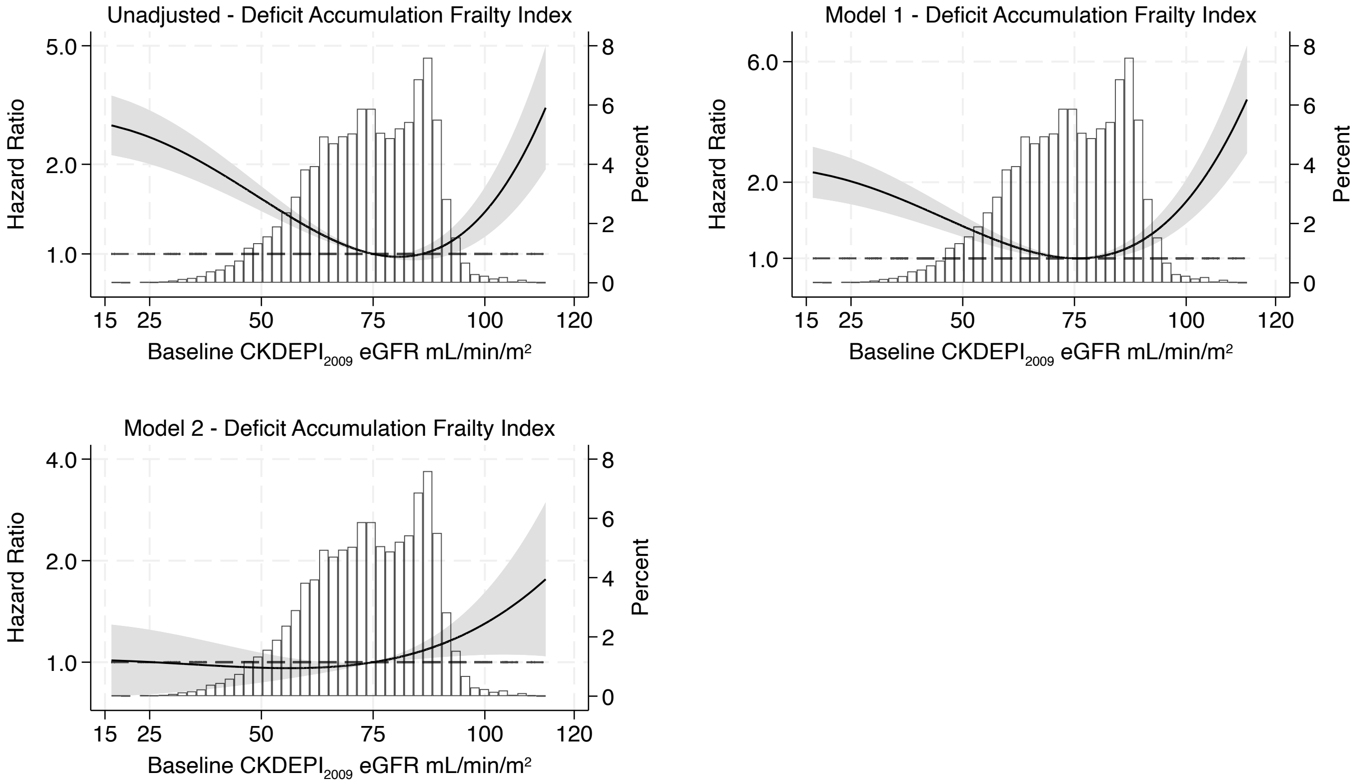


eGFR: estimated glomerular filtration rate; CKD-EPI: Chronic Kidney Disease Epidemiology Collaboration.

**Supplementary Figure S6.** Derivation of cohort for incident frailty defined using the deficit accumulation frailty index. Participants was excluded from analysis if they were frail at baseline, or if they had missing frailty data at baseline or longitudinally. A frailty index score of >0.21 was classified as 'frail'. Participants were also excluded if they were missing baseline kidney function measures (eGFR or UACR).  accumulation frailty index.

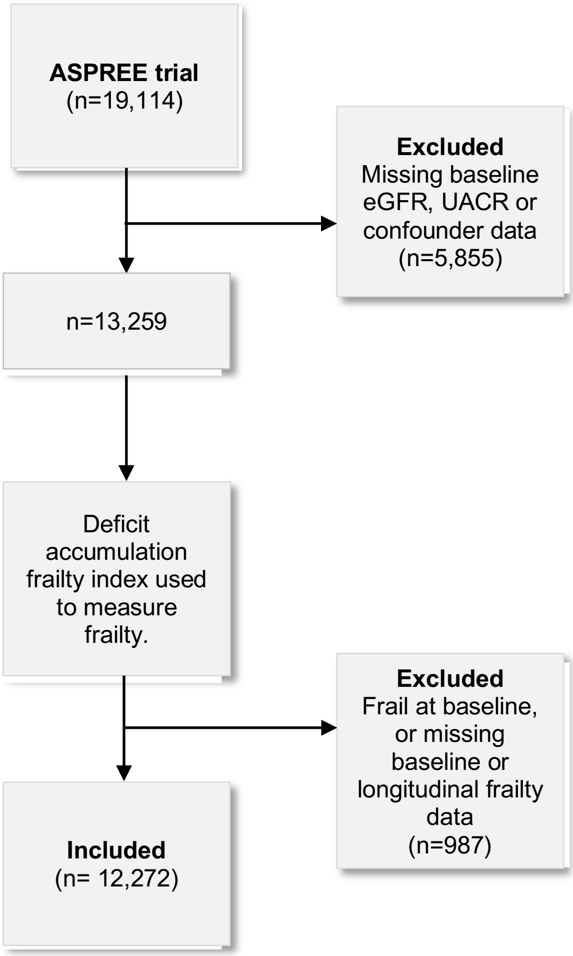


ASPREE: ASPirin in Reducing Events in the Elderly; eGFR: estimated glomerular filtration rate, UACR: urine albumin to creatinine ratio.

**Supplementary Table S1:** Number and percentage of participants with missing data for each of the Fried frailty phenotype components at each follow-up visit of the ASPREE trial.

| Follow-up visit | n | Slowness n(%) | Activity level n(%) | Exhaustion n(%) | Weight loss n(%) | Weakness n(%) |
|---|---|---|---|---|---|---|
| **Baseline** | 16,965 | 0 (0) | 0 (0) | 0 (0) | 0 (0) | 0 (0) |
| **Year 1** | 16,867 | 16,867 (100) | 757 (4) | 767 (5) | 1,077 (6) | 16,867 (100) |
| **Year 2** | 16,677 | 1716 (10) | 1,146 (7) | 10,458 (63) | 1,875 (11) | 1,852 (11) |
| **Year 3** | 14,716 | 13,689 (93) | 1,368 (9) | 1,397 (9) | 2,129 (14) | 13,709 (93) |
| **Year 4** | 11,267 | 1,693 (15) | 1,170 (10) | 2,132 (19) | 1,810 (16) | 1,800 (16) |
| **Year 5** | 7,276 | 6,022 (83) | 940 (13) | 956 (13) | 1,343 (18) | 6,029 (83) |
| **Year 6** | 2,688 | 636 (24) | 476 (18) | 486 (18) | 651 (24) | 666 (25) |
| **Year 7** | 81 | 34 (42) | 30 (37) | 30 (37) | 35 (43) | 81 (100) |

ASPREE: ASPirin in Reducing Events in the Elderly
Participants were followed up for a median of 4.7 years and attended clinic for annual study visits. As per the ASPREE study design protocol, not all measures relevant to the frailty phenotype were assessed at every visit such that the proportion of missing data for each frailty component varied from visit to visit.

**Supplementary Table S2.** Imputation methods.

| Imputed variable | Model | Predictor variables |
|---|---|---|
| **Education** | Logistic | country, age, gender, living, ethnic group, born overseas, language, hypertension (derived), diabetes(derived), smoking, alcohol |
| **Ethnic group** | Multinomial | country, age, gender, education, living, born overseas, language, hypertension (derived), diabetes (derived), smoking, alcohol |
| **Race** | Logistic | country, age, gender, born overseas, language |
| **Height** | PMM (GLM) | age, gender, weight |
| **Weight** | PMM (GLM) | ID, visit, age, gender, height |
| **Body mass index** | Passive | height, weight |
| **Dominant hand grip (one, two, three)** | PMM (GLMM) | ID, visit, country, age, gender, education, living, ethnic group, born overseas, language, hypertension, diabetes, smoking, alcohol, BMI, grip one (for grip two), grip two (for grip three) |
| **Grip mean** | Passive | Mean of grip one, grip two, grip three |
| **Grip group** | Passive | BMI, gender |
| **P20 grip** | Passive | Grip group, grip mean |
| **Weakness** | Passive | Grip mean, p20 grip distribution, mean of cluster (person) eGFR, mean of cluster (person) uACR |
| **Gait trial (one, two)** | PMM (GLMM) | ID, visit, country, age, gender, education, living, ethnic group, born overseas, language, hypertension (derived), diabetes (derived), smoking, alcohol, BMI, grip one, grip two, grip three, gait one (for gait two), gait two (for gait one) |
| **Gait mean** | Passive | mean of gait one, gait two |
| **Gait group** | Passive | height, gender |
| **P80 gait distribution** | Passive | gait group, gait mean |
| **Slowness** | Passive | gait mean, p80 gait distribution, mean of cluster (person) eGFR, mean of cluster (person) uACR |
| **Weight change** | Passive | weight, BMI |
| **Shrinkage** | Passive | weight change, BMI |
| **CES-D Q. 4 (everything an effort)** | PMM (GLMM) | ID, visit, age, gender, hypertension (derived), diabetes (derived), smoking, alcohol, BMI |
| **CES-D Q.10 (could not get going)** | PMM (GLMM) | ID, visit, age, gender, hypertension (derived), diabetes (derived), smoking, alcohol, BMI, CESD4, |
| **Exhaustion** | Passive | CES-D 4, CES-D 10, mean of cluster (person) eGFR, mean of cluster (person) uACR |
| **Walk outside** | PMM (GLMM) | ID, visit, age, gender, hypertension (derived), diabetes (derived), smoking, alcohol, BMI |
| **Walk time** | PMM (GLMM) | ID, visit, age, gender, hypertension (derived), diabetes (derived), smoking, alcohol, BMI, walk outside |
| **Low activity** | Passive | walk outside, walk time, mean of cluster (person) eGFR, mean of cluster (person) uACR |

eGFR: estimated glomerular filtration rate; UACR: urine albumin to creatinine ratio; CES-D: Centre for Epidemiological Studies Depression Scale; P20: 20$^{th}$ percentile; P80: 80$^{th}$ percentile; PMM: predictive mean matching, GLM: general linear model; GLMM: generalized linear mixed model.

**Supplementary Table S3.** Hazard Ratios describing the association between baseline estimated glomerular filtration rate, using the 2021 CKD-EPI equation, and risk of incident frailty.

| | Fried frailty phenotype* HR (95%CI) | | | Deficit accumulation frailty index HR (95%CI) | | |
|---|---|---|---|---|---|---|
| **eGFR categories (mL/min/1.73m$^2$)** | **Unadjusted** | **Adjusted 1[#]** | **Adjusted 2[##]** | **Unadjusted** | **Adjusted 1[#]** | **Adjusted 2[##]** |
| <45 | 2.25 (2.00-2.50) | 1.47 (1.23-1.72) | 1.12 (0.87-1.37) | 2.08 (1.65-2.62) | 1.73 (1.37-2.18) | 1.05 (0.83-1.33) |
| 45 to 59 | 1.48 (1.7-1.59) | 1.18 (1.05-1.31) | 1.00 (0.88-1.12) | 1.36 (1.20-1.55) | 1.23 (1.08-1.40) | 0.88 (0.77-1.00) |
| 60 to 89 | 1.00 | 1.00 | 1.00 | 1.00 | 1.00 | 1.00 |
| ≥90 | 0.86 (0.73-0.98) | 1.06 (0.97-1.16) | 1.09 (0.99-1.19) | 0.93 (0.84-1.03) | 1.08 (0.98-1.20) | 1.08 (0.97-1.20) |

HR: hazard ratio. CI: confidence interval. eGFR: estimated glomerular filtration rate calculated with the 2021 CKD-EPI equation; CKD-EPI: Chronic Kidney Disease Epidemiology Collaboration.
*Pooled estimates of 10 imputed datasets using Rubin's rules
[#] Model 1, adjusted for age and sex.
[##] Model 2, adjusted for age, sex, log2(urine albumin-to-creatinine ratio), smoking, alcohol, body mass index, education, polypharmacy, dyslipidaemia, ASPREE study treatment (aspirin or placebo), pre-frailty status, country of residence, diabetes, systolic blood pressure and diastolic blood pressure.

**Supplementary Table S4:** Hazard Ratios for baseline log2-transformed urine albumin-to-creatinine ratio in discrete time survival analysis of incident using the Fried frailty phenotype with observed, non-imputed data.

| **Unadjusted model** HR (95%CI) | **Adjusted Model 1**[#] HR (95%CI) | **Adjusted model 2**[##] HR (95%CI) |
|---|---|---|
| 1.17 (1.13-1.21), p<0.001 | 1.12 (1.07-1.16), p<0.001 | 1.05 (1.01-1.09), p=0.011 |

HR: hazard ratio. CI: confidence interval.
[#] Model 1, adjusted for age and sex.
[##] Model 2, adjusted for age, sex, estimated glomerular filtration rate, smoking, alcohol, body mass index, education, polypharmacy, dyslipidaemia, ASPREE study treatment (aspirin or placebo), baseline pre-frailty status, country of residence, diabetes, systolic blood pressure and diastolic blood pressure.

**Supplementary Table S5.** Hazard ratios for baseline estimated glomerular filtration rate in discrete time survival analysis of incident frailty, using the Fried frailty phenotype and observed, non-imputed data.

| **eGFR categories (mL/min/1.73m$^2$)** | **Unadjusted** HR (95%CI) | **Model 1#** HR (95%CI) | **Model 2##** HR (95%CI) |
|---|---|---|---|
| <45 | 2.11 (1.56-2.84) | 1.49 (1.10-2.02) | 1.05 (0.76-1.45) |
| 45 to 59 | 1.28 (1.07-1.54) | 1.06 (0.88-1.27) | 0.84 (0.69-1.02) |
| 60 to 89 | 1.00 | 1.00 | 1.00 |
| ≥90 | 0.85 (0.73-0.99) | 1.17 (1.00-1.38) | 1.17 (0.99-1.38) |

HR: hazard ratio. CI: confidence interval. eGFR: estimated glomerular filtration rate (calculated with 2021 CKD-EPI). CI: confidence interval; CKD-EPI: Chronic Kidney Disease Epidemiology Collaboration

*Pooled estimates of 10 imputed datasets using Rubin's rules

# Model 1, adjusted for age and sex.

## Model 2, adjusted for age, sex, log2(urine albumin-to-creatinine ratio), smoking, alcohol, body mass index, education, polypharmacy, dyslipidaemia, ASPREE study treatment (aspirin or placebo), pre-frailty status, country of residence, diabetes, systolic blood pressure and diastolic blood pressure.

**Supplementary Table S6:** Baseline characteristics of ASPREE participants overall and by baseline eGFR calculated with 2009 CKD-EPI value using the Fried frailty phenotype to measure frailty.

| | **Overall** (n=16,965) | **Baseline eGFR (mL/min/1.73m²)** | | | |
|---|---|---|---|---|---|
| | | **<45** (n=537) | **45-59** (n=2,532) | **60-89** (n=12,624) | **≥90** (n=1,272) |
| Age (years), mean (SD) | 75.0 (4.5) | 78.2 (5.5) | 76.7 (5.0) | 74.9 (4.2) | 72.1 (3.2) |
| Female, n (%) | 9,521 (56) | 324 (60) | 1,498 (59) | 6,944 (55) | 755 (59) |
| Country, n (%) | | | | | |
| Australia | 14,783 (87) | 466 (87) | 2,174 (86) | 11,218 (89) | 925 (73) |
| United States | 2,182 (13) | 741 (13) | 358 (14) | 1,406 (11) | 347 (27) |
| ASPREE treatment group, n (%) | | | | | |
| Aspirin | 8,432 (50) | 280 (52) | 1,273 (50) | 6,236 (49) | 643 (51) |
| Placebo | 8,533 (50) | 257 (48) | 1,259 (50) | 6,388 (51) | 629 (49) |
| Pre-frail [#], n (%) | 6,709 (40) | 301 (56) | 1,101 (43) | 4,751 (38) | 556 (44) |
| Education (years), n (%) | | | | | |
| Less than 12 | 7,790 (51) | 274 (56) | 1,207 (52) | 5,624 (49) | 496 (44) |
| 12 or more | 7,601 (49) | 215 (44) | 1,113 (48) | 5,825 (51) | 637 (56) |
| Smoking, n (%) | | | | | |
| Current | 648 (4) | 17 (3) | 86 (3) | 444 (4) | 101 (8) |
| Former | 6,951 (41) | 194 (36) | 1,034 (41) | 5,199 (41) | 523 (41) |
| Never | 9,366 (55) | 325 (61) | 1,412 (56) | 6,981 (55) | 648 (51) |
| Alcohol use, n (%) | | | | | |
| Current | 13,053 (77) | 356 (66) | 1,860 (73) | 9,880 (78) | 957 (75) |
| Former | 1,004 (6) | 42 (8) | 154 (6) | 703 (6) | 105 (8) |
| Never | 2,908 (17) | 139 (26) | 518 (20) | 2,041 (16) | 210 (17) |
| Body mass index (kg/m²), mean (SD) | 28 (4.7) | 29 (5.1) | 29 (4.7) | 28 (4.6) | 28 (5.2) |
| Body mass index, n (%) | | | | | |
| < 20 | 59 (0.3) | 2 (0.5) | 2 (<0.1) | 47 (0.4) | 8 (0.6) |
| 20-24.9 | 4,424 (26) | 99 (18) | 555 (22) | 3,404 (27) | 366 (29) |
| 25-29.9 | 7,543 (44) | 237 (44) | 1,163 (46) | 5,641 (45) | 502 (39) |
| 30-34.9 | 3,604 (21) | 133 (25) | 584 (23) | 2,608 (21) | 279 (22) |
| ≥35 | 1,335 (8) | 66 (12) | 228 (9) | 924 (7) | 117 (9) |
| Hypertension, n (%) | 12,586 (74) | 488 (91) | 2,089 (83) | 9,109 (72) | 900 (71) |
| Blood pressure (mmHg), mean (SD) | | | | | |
| Systolic | 139 (16) | 141 (17) | 140 (17) | 139 (16) | 138 (16) |
| Diastolic | 77 (10) | 76 (11) | 77 (10) | 77 (10) | 78 (9) |
| Diabetes mellitus, n (%) | 1,805 (11) | 93 (17) | 345 (14) | 1,188 (9) | 179 (14) |
| Polypharmacy, n (%) | 4,427 (26) | 263 (49) | 821 (32) | 3,015 (24) | 328 (26) |
| Dyslipidaemia, n (%) | 11,103 (65) | 380 (71) | 1,718 (68) | 8,226 (65) | 779 (61) |
| UACR (mg/mmol), median (IQR) | 0.8 (0.5, 1.5) | 1.1 (0.6, 3.3) | 0.8 (0.5, 1.8) | 0.8 (0.5, 1.4) | 0.9 (0.5, 1.8) |

Observed data; not imputed. ASPREE: ASPirin in Reducing Events in the Elderly; eGFR: estimated glomerular filtration rate; UACR: urine albumin to creatinine ratio; IQR: interquartile range; SD: standard deviation.; CKD-EPI: Chronic Kidney Disease Epidemiology Collaboration

[#] Pre-fail defined using the Fried Frailty Phenotype score. A score of 0 indicates no frailty, 1 or 2 is 'pre-frail' and 3, 4 or 5 is 'frail'.

**Supplementary Table S7:** Baseline characteristics of ASPREE participants included for analyses using the deficit accumulation frailty index to measure frailty, and the 2009 CKD-EPI equation for eGFR.

| | **Overall** n=12,308 | Baseline eGFR (mL/min/1.73m$^2$) | | | |
|---|---|---|---|---|---|
| | | **<45** (n=317) | **45-59** (n=1,731) | **60-89** (n=9,371) | **≥90** (n=889) |
| Age (years), mean (SD) | 74.8 (4.2) | 78.0 (4.3) | 76.3 (4.8) | 74.7 (4.1) | 72.1 (2.9) |
| Female, n (%) | 6,627 (54) | 178 (56) | 962 (56) | 4,965 (53) | 522 (59) |
| Country, n (%) | | | | | |
| Australia | 11,055 (09) | 282 (89) | 1,535 (89) | 8,539 (991) | 699 (79) |
| United States | 1,253 (10) | 35 (11) | 196 (11) | 832 (9) | 190 (21) |
| Ethnicity/Race | | | | | |
| White, Australian | 10,919 (89) | 276 (87) | 1,521 (88) | 8,433 (90) | 689 (78) |
| White, US | 677 (5) | 17 (5) | 119 (7) | 491 (5) | 50 (5) |
| African American | 429 (3) | 16 (5) | 61 (3) | 241 (3) | 111 (12) |
| Hispanic | 133 (1) | 2 (1) | 15 (1) | 92 (1) | 24 (3) |
| Other | 145 (2) | 6 (2) | 14 (1) | 110 (1) | 15 (2) |
| ASPREE treatment group, n (%) | | | | | |
| Aspirin | 6,081 (49) | 168 (53) | 844 (49) | 4,615 (49) | 545 (51) |
| Placebo | 6,227 (51) | 149 (47) | 887 (51) | 4,756 (51) | 435 (49) |
| Pre-frail, n (%) | 5,544 (45) | 230 (73) | 1,100 (64) | 3,832 (41) | 382 (43) |
| Education (years), n (%) | | | | | |
| Less than 12 | 5,949 (48) | 171 (54) | 874 (50) | 4,527 (48) | 377 (42) |
| 12 or more | 6,359 (52) | 146 (46) | 857 (50) | 4,844 (52) | 512 (58) |
| Smoking, n (%) | | | | | |
| Current | 414 (3) | 5 (1.6) | 54 (3) | 7,433 (79) | 682 (77) |
| Former | 4,964 (40) | 114 (36) | 706 (41) | 464 (5) | 77 (9) |
| Never | 6,930 (56) | 198 (62) | 971 (56) | 1,474 (16) | 130 (15) |
| Alcohol use, n (%) | | | | | |
| Current | 9,647 (78) | 216 (68) | 1316 (76) | 7433 (79) | 682 (77) |
| Former | 645 (5) | 15 (5) | 89 (5) | 464 (5) | 77 (9) |
| Never | 2,016 (16) | 86 (27) | 326 (19) | 1474 (16) | 130 (15) |
| Body mass index (kg/m$^2$), mean (SD) | 27.8 (4.4) | 28.6 (5.0) | 28.1 (4) | 27.7 (4) | 27.7 (5) |
| Body mass index, n (%) | | | | | |
| < 20 | 208 (1.7) | 4 (1) | 21 (1) | 154 (2) | 29 (4) |
| 20-24.9 | 3089 (25) | 61 (19) | 370 (21) | 2414 (26) | 244 (27) |
| 25-29.9 | 5713 (46) | 147 (46) | 846 (49) | 4352 (46) | 368 (41) |
| 30-34.9 | 2468 (20) | 71 (22) | 369 (21) | 1847 (20) | 181 (20) |
| ≥35 | 830 (7) | 34 (11) | 125 (7) | 604 (6) | 67 (8) |
| Hypertension, n (%) | 9007 (73) | 285 (90) | 1381 (80) | 6721 (72) | 620 (70) |
| Blood pressure (mmHg), mean (SD) | | | | | |
| Systolic | 139 (16) | 141 (18) | 140 (17) | 139 (16) | 138 (16) |
| Diastolic | 77 (10) | 76 (11) | 77 (10) | 77 (10) | 77 (9) |
| Diabetes mellitus, n (%) | 1194 (10) | 52 (16) | 206 (12) | 817 (9) | 119 (13) |
| Polypharmacy, n (%) | 2795 (23) | 128 (40) | 483 (28) | 1962 (21) | 222 (25) |
| Dyslipidaemia, n (%) | 4263 (35) | 105 (33) | 569 (33) | 3248 (35) | 341 (38) |
| UACR (mg/mmol), median (IQR) | 0.80 (0.40,1.40) | 1.00 (0.60,3.00) | 0.80 (0.50,1.60) | 0.80 (0.40,1.30) | 0.90 (0.50,1.70) |

Baseline demographic variables of participants who were not frail at time of entry to the ASPREE trial. Observed data; not imputed. ASPREE: ASPirin in Reducing Events in the Elderly; eGFR: estimated glomerular filtration rate (estimated with the 2009 CKD-EPI equation); UACR: urine albumin to creatinine ratio; IQR: interquartile range. SD: standard deviation; CKD-EPI: Chronic Kidney Disease Epidemiology Collaboration

**Supplementary Table S8:** Hazard Ratio for the association between baseline $log_2$-transformed baseline urine albumin-to-creatinine ratio and incident frailty using two measures of frailty. The 2009 CKD-EPI equation was used to calculate eGFR and classify CKD for use in the frailty index.

| | **Fried frailty phenotype** HR (95%CI)* | **Deficit accumulation frailty index** HR (95%CI) |
|---|---|---|
| **Unadjusted** | 1.10 (1.05-1.50), p=0.001 | 1.14 (1.11-1.17), p<0.001 |
| **Adjusted 1[#]** | 1.07 (1.03-1.10), p=0.002 | 1.10 (1.07-1.13), p<0.001 |
| **Adjusted 2[##]** | 1.04 (1.01-1.06), p=0.003 | 1.04 (1.01-1.06), p=0.014 |

HR: hazard ratio. CI: confidence interval. CKD-EPI: Chronic Kidney Disease Epidemiology Collaboration
*Pooled estimates of 10 imputed datasets using Rubin's rules
[#] Model 1, adjusted for age and sex.
[##] Model 2, adjusted for age, sex, estimated glomerular filtration rate, smoking, alcohol, body mass index, education, polypharmacy, dyslipidaemia, ASPREE study treatment (aspirin or placebo), pre-frailty status, country of residence, diabetes, systolic blood pressure and diastolic blood pressure.
Estimated glomerular filtration rate calculated using 2009 CKD-EPI equation.

**Supplementary Table S9.** Hazard Ratios describing the association between baseline estimated glomerular filtration rate, using 2009 CKD-EPI equation, and risk of incident frailty.

| | **Fried frailty phenotype*** HR (95%CI) | | | **Deficit accumulation frailty index** HR (95%CI) | | |
|---|---|---|---|---|---|---|
| **eGFR categories ($mL/min/1.73m^2$)** | **Unadjusted** | **Adjusted 1#** | **Adjusted 2##** | **Unadjusted** | **Adjusted 1#** | **Adjusted 2##** |
| <45 | 2.15 (1.99-2.32) | 1.40 (1.21-1.59) | 1.01 (0.85-1.17) | 1.90 (1.55-2.32) | 1.48 (1.20-1.82) | 0.94 (0.76-1.15) |
| 45 to 59 | 1.41 (1.24-1.58) | 1.14 (1.03-1.24) | 0.97 (0.84-1.10) | 1.43 (1.28-1.59) | 1.27 (1.14-1.42) | 0.91 (0.82-1.02) |
| 60 to 89 | 1.00 (reference) | 1.00 (reference) | 1.00 (reference) | 1.00 (reference) | 1.00 (reference) | 1.00 (reference) |
| ≥90 | 1.31 (1.03-1.59) | 1.84 (1.66-2.01) | 1.43 (1.24-1.63) | 1.16 (0.99-1.36) | 1.35 (1.15-1.58) | 1.21 (1.03-1.42) |

HR: hazard ratio. CI: confidence interval. eGFR: estimated glomerular filtration rate (calculated using 2009 CKD-EPI equation); CKD-EPI: Chronic Kidney Disease Epidemiology Collaboration

*Pooled estimates of 10 imputed datasets using Rubin's rules

# Model 1, adjusted for age and sex.

## Model 2, adjusted for age, sex, log2(urine albumin-to-creatinine ratio), smoking, alcohol, body mass index, education, polypharmacy, dyslipidaemia, ASPREE study treatment (aspirin or placebo), pre-frailty status, country of residence, diabetes, systolic blood pressure and diastolic blood pressure.

**Supplementary Table S10.** Hazard ratios for baseline eGFR using 2009 CKD-EPI in discrete time survival analysis of incident frailty, using the Fried frailty phenotype and observed, non-imputed data.

| **eGFR categories (mL/min/1.73m$^2$)** | **Unadjusted** HR (95%CI) | **Model 1#** HR (95%CI) | **Model 2##** HR (95%CI) |
|---|---|---|---|
| <45 | 2.01 (1.55-2.61) | 1.25 (0.95-1.63) | 0.89 (0.67-1.17) |
| 45 to 59 | 1.24 (1.05-1.46) | 0.98 (0.83-1.15) | 0.84 (0.71-0.99) |
| 60 to 89 | 1.00 (reference) | 1.00 (reference) | 1.00 (reference) |
| ≥90 | 1.38 (1.12-1.71) | 2.01 (1.61-2.50) | 1.59 (1.25-2.01) |

HR: hazard ratio. CI: confidence interval. eGFR: estimated glomerular filtration rate. CI: confidence interval; CKD-EPI: Chronic Kidney Disease Epidemiology Collaboration
*Pooled estimates of 10 imputed datasets using Rubin's rules
# Model 1, adjusted for age and sex.
## Model 2, adjusted for age, sex, log2(urine albumin-to-creatinine ratio), smoking, alcohol, body mass index, education, polypharmacy, dyslipidaemia, ASPREE study treatment (aspirin or placebo), pre-frailty status, country of residence, diabetes, systolic blood pressure and diastolic blood pressure.

# Reporting checklist for cohort study.

Based on the STROBE cohort guidelines.

| | | Reporting Item | Page Number |
|---|---|---|---|
| **Title and abstract** | | | |
| Title | #1a | Indicate the study's design with a commonly used term in the title or the abstract | 1 |
| Abstract | #1b | Provide in the abstract an informative and balanced summary of what was done and what was found | 3 |
| **Introduction** | | | |
| Background / rationale | #2 | Explain the scientific background and rationale for the investigation being reported | 4 |
| Objectives | #3 | State specific objectives, including any prespecified hypotheses | 5 |
| **Methods** | | | |
| Study design | #4 | Present key elements of study design early in the paper | 6 |
| Setting | #5 | Describe the setting, locations, and relevant dates, including periods of recruitment, exposure, follow-up, and data collection | 6 |
| Eligibility criteria | #6a | Give the eligibility criteria, and the sources and methods of selection of participants. Describe methods of follow-up. | 6 |
| Eligibility criteria | #6b | For matched studies, give matching criteria and number of exposed and unexposed | 6 |
| Variables | #7 | Clearly define all outcomes, exposures, predictors, potential confounders, and effect modifiers. Give diagnostic criteria, if applicable | 7 |
| Data sources / measurement | #8 | For each variable of interest give sources of data and details of methods of assessment (measurement). Describe comparability of assessment methods if there is more than one group. Give information separately for for exposed and unexposed groups if applicable. | 6-7 |
| Bias | #9 | Describe any efforts to address potential sources of bias | 7-11 |
| Study size | #10 | Explain how the study size was arrived at | 6,12 |
| Quantitative variables | #11 | Explain how quantitative variables were handled in the analyses. If applicable, describe which groupings were chosen, and why | 7-11 |
| Statistical methods | #12a | Describe all statistical methods, including those used to control for confounding | 7-11 |
| Statistical methods | #12b | Describe any methods used to examine subgroups and interactions | n/a |
| Statistical methods | #12c | Explain how missing data were addressed | 8-9 |
| Statistical methods | #12d | If applicable, explain how loss to follow-up was addressed | n/a |
| Statistical methods | #12e | Describe any sensitivity analyses | 11 |
| **Results** | | | |
| Participants | #13a | Report numbers of individuals at each stage of study—eg numbers potentially eligible, examined for eligibility, confirmed eligible, included in the study, completing follow-up, and analysed. Give information separately for for exposed and unexposed groups if applicable. | 12,14 |
| Participants | #13b | Give reasons for non-participation at each stage | 12,14 |
| Participants | #13c | Consider use of a flow diagram | Supp. File |
| Descriptive data | #14a | Give characteristics of study participants (eg demographic, clinical, social) and information on exposures and potential confounders. Give information separately for exposed and unexposed groups if applicable. | 12,15, Table 1, Table 4 |
| Descriptive data | #14b | Indicate number of participants with missing data for each variable of interest | 13,15 |
| Descriptive data | #14c | Summarise follow-up time (eg, average and total amount) | 14,15 |

| | | | |
|---|---|---|---|
| Outcome data | #15 | Report numbers of outcome events or summary measures over time. Give information separately for exposed and unexposed groups if applicable. | 14 |
| Main results | #16a | Give unadjusted estimates and, if applicable, confounder-adjusted estimates and their precision (eg, 95% confidence interval). Make clear which confounders were adjusted for and why they were included | 12-15, Tables 2 and 3 |
| Main results | #16b | Report category boundaries when continuous variables were categorized | 12-15, Tables 2 and 3 |
| Main results | #16c | If relevant, consider translating estimates of relative risk into absolute risk for a meaningful time period | 13-16 |
| Other analyses | #17 | Report other analyses done—eg analyses of subgroups and interactions, and sensitivity analyses | 13,14 |
| **Discussion** | | | |
| Key results | #18 | Summarise key results with reference to study objectives | 16 |
| Limitations | #19 | Discuss limitations of the study, taking into account sources of potential bias or imprecision. Discuss both direction and magnitude of any potential bias. | 19, 20 |
| Interpretation | #20 | Give a cautious overall interpretation considering objectives, limitations, multiplicity of analyses, results from similar studies, and other relevant evidence. | 20 |
| Generalisability | #21 | Discuss the generalisability (external validity) of the study results | 19,20 |
| **Other Information** | | | |
| Funding | #22 | Give the source of funding and the role of the funders for the present study and, if applicable, for the original study on which the present article is based | 21 |

**Supplementary References**

S1. Delgado C, Baweja M, Crews DC, et al. A Unifying Approach for GFR Estimation: Recommendations of the NKF-ASN Task Force on Reassessing the Inclusion of Race in Diagnosing Kidney Disease. *American Journal of Kidney Diseases*. 2022;79(2):268-288.e1. doi:10.1053/j.ajkd.2021.08.003

S2. Ernst ME, Fravel MA, Webb KL, et al. Long-Term Blood Pressure Variability and Kidney Function in Participants of the ASPREE Trial. *Am J Hypertens*. 2022;35(2):173-181. doi:10.1093/ajh/hpab143

S3. Polkinghorne KR, Wetmore JB, Thao LTP, et al. Effect of Aspirin on CKD Progression in Older Adults: Secondary Analysis From the ASPREE Randomized Clinical Trial. *American Journal of Kidney Diseases*. 2022;80(6):810-813. doi:10.1053/j.ajkd.2022.02.019

S4. Fried LP, Tangen CM, Walston J, et al. Frailty in Older Adults: Evidence for a Phenotype. *J Gerontol A Biol Sci Med Sci*. 2001;56(3):M146-M157. doi:10.1093/gerona/56.3.M146

S5. Ryan J, Espinoza S, Ernst ME, et al. Validation of a Deficit-Accumulation Frailty Index in the ASPirin in Reducing Events in the Elderly Study and Its Predictive Capacity for Disability-Free Survival. *The Journals of Gerontology: Series A*. 2022;77(1):19-26. doi:10.1093/gerona/glab225
